# An artificial synapse based on molecular junctions

**DOI:** 10.1038/s41467-023-35817-5

**Published:** 2023-01-16

**Authors:** Yuchun Zhang, Lin Liu, Bin Tu, Bin Cui, Jiahui Guo, Xing Zhao, Jingyu Wang, Yong Yan

**Affiliations:** 1 CAS Key Laboratory of Nanosystem and Hierarchical Fabrication, CAS Center for Excellence in Nanoscience, National Center for Nanoscience and Technology, Beijing, 100190 China; 2 University of Chinese Academy of Sciences, Beijing, 100049 China; 3 School of Physics, Shandong University, Jinan, 250100 China; 4 Department of Chemistry, School of Chemistry and Biological Engineering, University of Science and Technology Beijing, Beijing, 100083 China

**Keywords:** Electronic devices, Electron transfer

## Abstract

Shrinking the size of the electronic synapse to molecular length-scale, for example, an artificial synapse directly fabricated by using individual or monolayer molecules, is important for maximizing the integration density, reducing the energy consumption, and enabling functionalities not easily achieved by other synaptic materials. Here, we show that the conductance of the self-assembled peptide molecule monolayer could be dynamically modulated by placing electrical biases, enabling us to implement basic synaptic functions. Both short-term plasticity (e.g., paired-pulse facilitation) and long-term plasticity (e.g., spike-timing-dependent plasticity) are demonstrated in a single molecular synapse. The dynamic current response is due to a combination of both chemical gating and coordination effects between Ag^+^ and hosting groups within peptides which adjusts the electron hopping rate through the molecular junction. In the end, based on the nonlinearity and short-term synaptic characteristics, the molecular synapses are utilized as reservoirs for waveform recognition with 100% accuracy at a small mask length.

## Abstract (web summary)

Designing scaled electronic devices for neuromorphic applications remains a challenge. Here, Zhang et al. develop an artificial molecular synapse based on self-assembled peptide molecule monolayer whose conductance can be dynamically modulated and used for waveform recognition.

## Introduction

The field of molecular electronics has been around for a half-century, but only recently has made some progress due to the development of robust wiring techniques^1,2^ and understanding of transport mechanisms^3–5^. Building electronic components using individual or monolayer molecules could be one of the solutions for the fundamental limitations that current complementary metal-oxide semiconductor (CMOS) technology faces upon further device miniaturization. Molecular electronic components such as switch^6^, rectifier (cf. diode)^7^, transistor^8^, memory^9,10^, and basic logic gates^11,12^ have been created. The development of molecular electronics is still on its way to replacing silicon electronics in which the information is processed based on the von Neumann architecture. However, this architecture separates the computation and memory steps which face great challenges upon explosive growth of data.

Neuromorphic computing, mimicking the information processing of the brain, could perform complex tasks with massive parallelism and work under Compute-in-Memory mode (*pace* von Neumann model), thus greatly improving both the computation and energy efficiencies^13–15^. Hardware implementation requires an artificial synapse that could emulate the change of connection strength (cf. synapse weight) between two processing neuros^16,17^. The electronic artificial synapse in which the synapse weight (cf. conductance) is modulated by electrical stimulus has attracted increasing attention since the seminal works of Leon Chua^18^ and Hewlett Packard team^19^ on their finding of memory resistors (memristor). In particular, the electric-field-induced memory phenomena have also been demonstrated by using molecular junctions as components in which the switching of resistance or conductance is dominated by either inherent characteristics of molecules^20–24^ or electrode-molecule couplings^25,26^. For example, the redox states^10,23^ and conformations^27^ of molecules could be electrically reconfigured, leading to drastically different electronic characters and reversible switching of conductance between two or multiple states. The discrete conductance states and their nonvolatile characteristics are both needed for fabricating large-scale, high-density molecular resistive random-access memory (RRAM) circuits, however, such digital switching performance is not capable of emulating synaptic functions in which the weight changes of molecular junction should be continuously modulated upon electrical pulses.

In this article, we report a molecular electronic synapse by sandwiching a self-assembled monolayer (SAM) of peptide (11 amino acids) molecules between an active Ag/AgO_*x*_ electrode and a eutectic alloy of Gallium and Indium (EGaIn) liquid electrode. The electric-field-driven pumping and migrating of Ag^+^ in peptide molecules could dynamically control the current passing across the junction, enabling us to modulate the synaptic weight and implement typical short-term and long-term synaptic plasticity (STP and LTP). To the best of our knowledge, this is the first experimental demonstration of a molecular artificial synapse within one molecule length scale. This molecular synapse is subsequently used in reservoir computing (RC) systems for recognizing simple sine and square waveforms.

## Results

### Molecular junction synapse

**Fig. 1:**
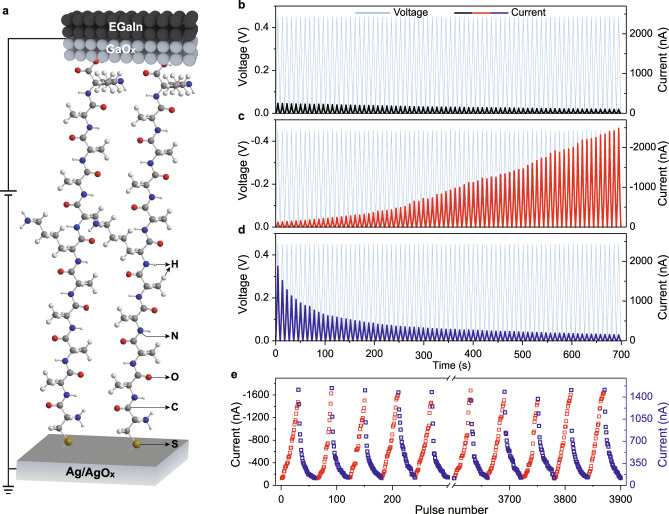
Molecular synapse architecture and dynamic current–voltage characteristics. **a** Scheme of the molecular synapse device containing a bottom Ag/AgO_*x*_ electrode, peptide SAM, and a liquid GaO_*x*_/EGaIn top electrode, Ag/AgO_*x*_//CAAAAKAAAAK//GaO_*x*_/EGaIn. The Ag/AgO_*x*_ electrode is prepared by thermal annealing electron-beam evaporated Ag layer (200 nm) at 150 °C for 40 min in air. Current signals (*black*, *red*, and *blue* curves) recorded by consecutively placing 75 positive **b**, negative **c**, and positive **d**, triangle pulses (*light blue* curves, amplitude, 0.45 V, width, 9 s). The bottom Ag/AgO_*x*_ is grounded. **e** Device endurance test by placing continuous potentiation (amplitude, −0.45 V, *red* markers, *left* *y*-axis) and depression (amplitude, 0.45 V, *blue* markers, *right* *y*-axis) pulses for 65 cycles. 30 square pulses (width, 0.5 s) are placed for each potentiation or depression.

The mission to continuously change the conductance of a molecular junction is largely impossible since there are very limited redox states or conformations of the molecule itself. Inspired by the influx and extrusion of Ca^2+^ in the biological synapse, an active electrode is needed, acting as a reservoir for donating/accepting metal cations into/from molecules. In addition, the molecules should contain functional groups which could interact with metal cations, enabling long-distance migration of cations^28^. Based on these two requirements, we design the device by using an active Ag/AgO_*x*_ electrode, an EGaIn (which consists of an ultrathin, highly conductive GaO_*x*_ layer on the surface) liquid electrode, and a monolayer of peptide molecules stuck in between, Ag/AgO_*x*_//peptide//GaO_*x*_/EGaIn (Fig. 1a, Supplementary Fig. 1, and see Supplementary Figs. 2–8 for the characterization of the junction). The Ag/AgO_*x*_ electrode is prepared by partial oxidation of electron-beam evaporated Ag in the air (see “Methods” section for details). This procedure decreases the energy needed for injecting Ag^+^ into peptide molecules (see below). The peptide molecule contains 11 amino acids (CAAAAKAAAAK, C, A, and K are cysteine, alanine, and lysine) terminated with cysteine, which provides thiol for bonding on the Ag surface. No bonding presents at the top liquid electrode but only physical contact.

The electrical characteristics of the molecular junction are then monitored by placing consecutive triangle pulses. When a string of positive pulses is firstly placed on the liquid electrode (the bottom Ag/AgO_*x*_ electrode is grounded), no apparent change of current is found (Fig. 1b). The slight decrease of current could be probably attributed to the anodic growth of the GaO_*x*_ layer, reorganization of the SAMs, and deformation of the PDMS top electrodes^29,30^. However, during the following sequence of negative pulses (with the same amplitude but reversed polarity), there is a dramatic enhancement of current, approximately 20 times increase after 75 triangular pulses (Fig. 1c). Interestingly, the high conductive states could return to the initial low conductive state under another sequence of consecutive positive pulses (Fig. 1d).

Apparently, this field polarity-dependent conductance modulation should be related to the active Ag/AgO_*x*_ electrode. We propose that the active Ag/AgO_*x*_ electrode has released Ag^+^ which enters into the peptide monolayer under negative pulses. This hypothesis is consistent with the artificial synapse or RRAM devices fabricated by using other interlayers^31^. The influx and extrusion (under positive pulses) of Ag^+^ are very similar to the behaviors of Ca^2+^ in bio-synapse. Accordingly, the increase and decrease of conductance (weight) correspond to the emulated synaptic potentiation (Fig. 1c and Supplementary Fig. 9, *red* half) and depression (Fig. 1d and Supplementary Fig. 9, *blue* half). Importantly, this continuous potentiation and depression process could be reversibly switched for up to tens of cycles within a single molecular synapse without apparent performance degradation (Fig. 1e and Supplementary Fig. 10).

### Operation mechanism of the molecular synapse

To clarify the dynamics of Ag^+^ within peptide SAMs, the performance of the active Ag/AgO_*x*_ electrode is firstly compared with the Ag electrode without thermal annealing (see Supplementary Fig. 5, Supplementary Fig.6, and Supplementary Fig. 11). In previous reports, stable electrical characteristics of Ag//SAM//GaO_*x*_/EGaIn devices are demonstrated, indicating no Ag^+^ release from the Ag electrode. Supplementary Fig. 6 compares the cyclic voltammetry (CV) curves of peptide SAM covalently bonded on Ag (*red*) and Ag/AgO_*x*_ (*blue*) electrodes. A pair of redox peaks around 0.3 and 0.2 V appears for Ag/AgO_*x*_//peptide device while no apparent peaks are found by using the Ag electrode. This is probably due to the different activation energies required for Ag^+^ release. Similar consecutive triangle pulses with an amplitude of 1.0 V are placed on the Ag//CAAAAKAAAAK//GaO_*x*_/EGaIn device (see Supplementary Fig. 11a–c). Neither apparent increase (potentiation) nor decrease (depression) in conductance are observed (Supplementary Fig. 11d–j), showing distinct electrical characteristics from Ag/AgO_*x*_//CAAAAKAAAAK//GaO_*x*_/EGaIn synapse (see Fig. 1 and Supplementary Fig. 11r–x). In another experiment, peptide SAM is replaced by using a monolayer of dodecanethiol, Ag/AgO_*x*_///HS-(CH_2_)_12_H//GaO_*x*_/EGaIn. The current–voltage characteristics of this dodecanethiol junction are analogous to that recorded in Ag//CAAAAKAAAAK//GaO_*x*_/EGaIn device (Supplementary Fig. 11k–q). The absence of conductance modulation in these two control devices indicates that both Ag/AgO_*x*_ active electrode and peptide molecules are indispensable for emulating synapses.

The roles of molecules and metal cations on the synaptic behaviors of the junctions are subsequently studied by performing several additional control experiments. Supplementary Fig. 12 shows the electrical characteristics of the Ag/AgO_*x*_//C(GABA)(GABA)D(GABA)(GABA)D//GaO_*x*_/EGaIn and Ag/AgO_*x*_//HS-PEG8-CH_2_CH_2_COOH//GaO_*x*_/EGaIn devices. Both devices exhibit similar synaptic behavior to the Ag/AgO_*x*_//CAAAAKAAAAK//GaO_*x*_/EGaIn junction, however, with relatively lower current change ratios (calculated by Ratio = *I*_80_/*I*_1_, where *I*_80_ (*I*_1_) is the measured current peak of the 80th (1st) pulse) under the same potentiation pulse sequences (Supplementary Fig. 11w vs. Supplementary Fig. 12d and Fig. 12h). On the other hand, the electrical characteristics of the Ag//CAAAAKAAAAK//GaO_x_/EGaIn junctions doped with Ag^+^, Na^+^, and Ca^2+^ cations (prepared by immersing Ag//CAAAAKAAAAK in AgNO_3_, NaNO_3_ and Ca(NO_3_)_2_ solution, see Methods section for details) are recorded (Supplementary Fig. 13). All of the cation-doped junctions display a conductance depression in the first sequence of positive pulses which is distinct from the device without metal cation doping (Supplementary Fig. 13 vs. Supplementary Fig. 11d). Interestingly, in the following sequences of negative and positive pulses, the devices show similar synaptic potentiation and depression behaviors. The current change ratios are positively related to the metal cation concentrations. Importantly, since the Na^+^ or Ca^2+^ ions are hard to be reduced into the metallic states (amplitude, 1 V), the synaptic properties in these devices are not due to the formation of the metallic filaments^31^. Instead, it should be attributed to the influence of cations on the electronic states of the peptide molecules. Finally, the temperature-dependent conductance measurements of Ag//CAAAAKAAAAK//GaO_*x*_/EGaIn and Ag//CAAAAKAAAAK-Ag^+^//GaO_*x*_/EGaIn (see “Methods” section for details) devices are performed. The activation energy has decreased from 128 to 36 meV upon binding of Ag^+^ (see Supplementary Fig. 14), indicating a higher electron hopping rate within the peptide-Ag^+^ junction.

To validate the reproducibility of our devices, a similar statistical analysis with Whitesides and Nijhuis reports is given in Supplementary Fig. 15 and Table S1. The counts of log_10_|*J*| at 0.5 V are calculated from the *I*–*V* curves at the stable states. These current density counts of all devices exhibit tight gaussian distributions^32,33^. In addition, the measurement of HS-(CH_2_)_12_H SAMs (<log_10_|*J*| > ~−2) is consistent with the previous results^33,34^. The conductance of HS-PEG8-CH_2_CH_2_COOH (<log_10_|*J*| > ~−1.7) is lower than the Au/HS-PEG8-CH_3_//GaOx/EGaIn junction (<log_10_|*J*| > ~−1.2)^32^, which is probably due to the different terminal groups and/or defects in the SAMs^35^. The junctions of C(GABA)(GABA)D(GABA)(GABA)D (<log_10_|*J*| > ~−3.1) and CAAAAKAAAAK (<log_10_|*J*| > ~−2.5) SAMs show similar conductance, which are larger than the value observed in Cys(Gly)_9_ SAM (<log_10_|*J*| > ~−5.4) with similar molecular backbones^32^. Compared to the Cys(Gly)_9_, the CAAAAKAAAAK molecules assembled on the electrode surface are much more ordered and have a larger tilt angle, which may lead to stronger couplings between the CAAAAKAAAAK and the electrode and enhance the electron transport in the molecular junction.

**Fig. 2:**
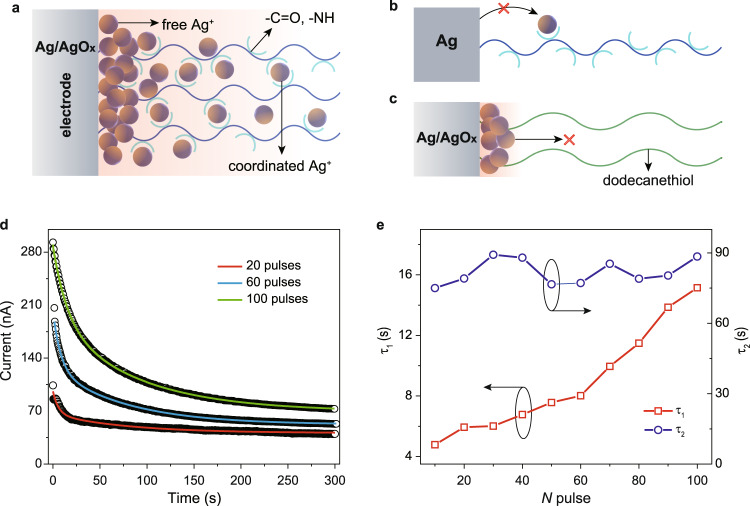
Operation mechanism of the molecular synapse. **a** Schematic showing the release and distribution of Ag^+^ in Ag/AgO_x_//CAAAAKAAAAK//GaO_*x*_/EGaIn device under negative voltage. The coordination between Ag^+^ and –C=O, –NH groups enables the long-distance transport of silver cations. **b** Ag^+^ couldn’t be activated in Ag//CAAAAKAAAAK//GaO_*x*_/EGaIn device. Much higher energy is required. **c** Ag^+^ couldn’t migrate across the long alkyl junction in Ag/AgO_*x*_///HS-(CH_2_)_12_H//GaO_*x*_/EGaIn device due to the lack of cation bridges. **d** Current decay curves of Ag/AgO_*x*_//CAAAAKAAAAK//GaO_*x*_/EGaIn molecular synapse after potentiated by 20, 60, and 100 square pulses (amplitude, −1 V). The colored solid lines are fitted curves with a double exponential function *I* = *a* ∗ exp (−*t*/*τ*_1_) + *b* ∗ exp (−*t*/*τ*_2_*)* *+* *c* in which the time constant *τ*_1_ and *τ*_2_ correspond to the fast and slow decaying process. **e** Dependence of the fitting time constants *τ*_1_ and *τ*_2_ on the number of potentiation pulses. The amplitude is −1 V.

All control experiments suggest the injection of Ag^+^ from the Ag/AgO_*x*_ active electrode into the peptide monolayer which influences the electron transport through the peptide molecules. Fig 2a shows the scheme of possible Ag^+^ release and distribution within the Ag/AgO_*x*_//CAAAAKAAAAK//GaO_*x*_/EGaIn synapse device under negative bias. The positively charged Ag^+^ could not only coordinate to the –C=O, –NH groups (coordination effect) but also influence the electronic states of functional groups (chemical gating) within peptide molecules^36^. However, in Ag//CAAAAKAAAAK//GaO_*x*_/EGaIn device, the Ag^+^ is hard to inject into the peptide monolayer (Fig. 2b), probably due to the much higher activation energy required for the Ag electrode (see Supplementary Fig. 6). For Ag/AgO_*x*_///HS-(CH_2_)_12_H//GaO_*x*_/EGaIn device, electric field could inject the Ag^+^ whereas these Ag^+^ are not able to migrate across the long alkyl junction due to the lack of cation bridges (e.g., polar groups, see Fig. 2c). In addition, the effective sites assisting electron hopping are dependent not only on the structure of junction molecules (Supplementary Fig. 11 and Supplementary Fig. 12) but also on the concentration of metal cations (Supplementary Fig. 13).

To better understand the chemical gating (by free Ag^+^) and coordination effects, the current decay of Ag/AgO_*x*_//CAAAAKAAAAK//GaO_*x*_/EGaIn molecular synapse after potentiation (up to 100 pulses) is monitored. The depression curves could be fitted by using a double exponential function *I* = *a* ∗ exp (−*t*/*τ*_1_) + *b* ∗ exp (−*t*/*τ*_2_) + *c* in which *τ*_1_ and *τ*_2_ correspond to the fast and slow decaying process (Fig. 2d and Supplementary Fig. 16). Interestingly, the *τ*_1_ increases with the number of potentiation pulse while *τ*_2_ with a relatively larger value (~80 s) is independent on the pulse number (Fig. 2e). We propose that the *τ*_1_ and *τ*_2_ correspond to the two different binding interactions between Ag^+^ and functional groups on the peptide. The weak binding with smaller *τ*_1_ is attributed to the electrostatic interaction (chemical gating) from free Ag^+^. The quasi-linear increase with pulse number indicates a similar increase in free Ag^+^ concentration. *τ*_2_ corresponds to the coordination effect in which the stronger binding interaction requires a longer time for depression.

### Theoretical model

**Fig. 3:**
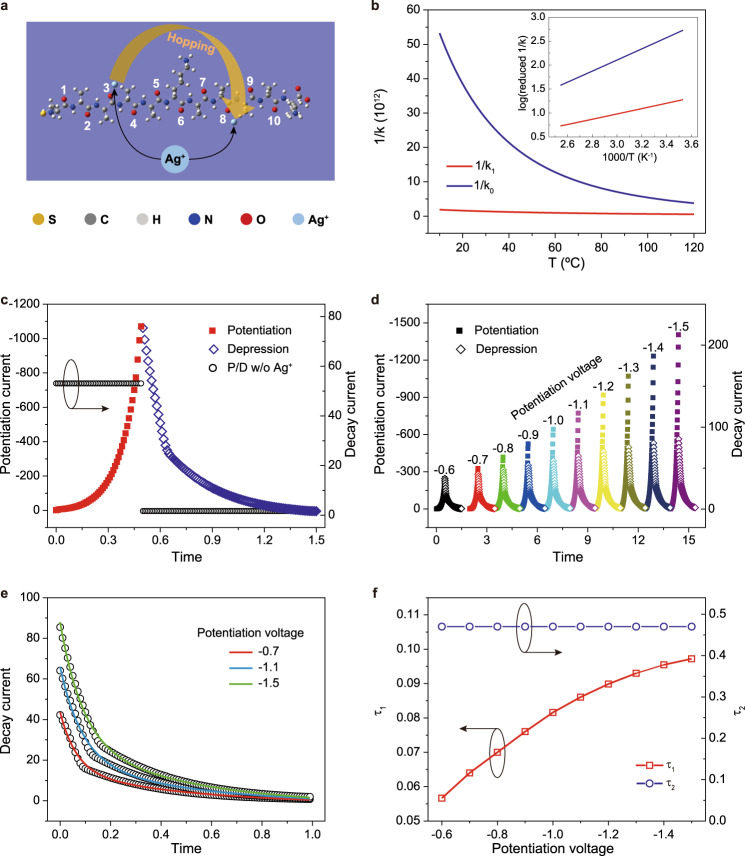
Modeling of the molecular synapse. **a** The schematic picture of the peptide with two Ag^+^ cations. **b** The inversed hopping rates of the peptides with (*red* curves) and without (*blue* curves) binding Ag^+^ were calculated based on the Marcus hopping theory. The inset shows the log(reduced 1/*k*) along with the inverted temperature (1000/T). **c** Simulated potentiation (*red* square, *left* *y*-axis) and depression (*blue* diamond, *right* *y*-axis) of Ag/AgO_*x*_//CAAAAKAAAAK//GaO_*x*_/EGaIn synapse. The Ag^+^ is injected by placing −1.3 V for 0.5 s while the depression process is monitored by placing a reversed 0.1 V for 1 s. The same voltages are placed on a control device (w/o Ag^+^), and no potentiation/depression characteristic is found (*black* circle, *right* *y*-axis). **d** Simulated potentiation (*solid* squares, *left* *y*-axis) and depression (*hollow* diamonds, *right* *y*-axis) of Ag/AgO_*x*_//CAAAAKAAAA //GaO_*x*_/EGaIn synapse under voltages from −0.6 to −1.5 V. **e** Simulated current decay curves after potentiated by −0.7, −1.1, and −1.5 V (*hollow* circles). The fitted solid lines are obtained by using a double exponential function $I=a*{{\exp }}\left(-t/{\tau }_{1}\right)+b*{{\exp }}\left(-t/{\tau }_{2}\right)+c$. **f** Dependence of the fitting time constants *τ*_1_ and *τ*_2_ on the potentiation voltage.

The influence of injected Ag^+^ cations upon electron transport and the synaptic potentiation/depression behavior is next studied by theoretical modeling. The structure of Ag//CAAAAKAAAAK//GaO_*x*_/EGaIn is optimized by the density functional theory (DFT) method. In this model, the peptides are anchored by sulfur atoms at the hollow positions of the $4\times 4$ supercells of the Ag (111) surfaces. A $7\times 7\times 1$ Monkhorst-Pack *k-*point^37^ sampling is used to treat the on-surface periodic characteristics of the peptide. The exchange-correlation potential is described by Perdew-Burke-Ernzerhof generalized gradient approximation (PBE-GGA)^38^. The core electrons are modeled with Troullier–Martins nonlocal pseudopotential^39^, and the valence electrons are expanded in a double-zeta localized (DZP) basis set^40^. All atoms in the unit cell are relaxed until the forces are smaller than 0.05 eV/Å. On the one hand, we perform a set of quantum chemical calculations by using Gaussian 16 package^41^ at B3LYP/6-311G* level^42,43^ and find a remarkable gap of about 5 eV between the highest occupied molecular orbital (HOMO) and the lowest unoccupied molecular orbital (LUMO). For simplicity, we only consider the HOMO and HOMO-1 as the hopping states for the bare peptide, which localize on the C and middle-chain K units, respectively. On the other hand, we adopt the wB97xd^44^ functional and Lanl2dz basis set^45^ to treat the system of peptide-Ag^+^. Each Ag^+^ induces an extra state in the HOMO-LUMO gap, promoting charge transfer. Based on the full geometry optimizations, Ag^+^ energetically favors adsorbing on the oxygen in the peptide chain with similar energies, as shown in Fig. 3a. Therefore, we randomly select the positions, e.g., No. 3 and 8, to examine the hopping processes. The hopping parameters^46,47^ are carried out and listed in Table S2. The calculated *E*_a_ of the peptide-Ag^+^ case is smaller than the bare case, which qualitatively agrees with the experimental results. Next, according to the equation ${k}_{{{{{{\rm{et}}}}}}}=\frac{2\pi }{h}\frac{{V}^{2}}{\sqrt{4\pi \lambda {k}_{{{{{{\rm{B}}}}}}}T}}{{{{{\rm{e}}}}}}^{-\frac{{E}_{a}}{{k}_{{{{{{\rm{B}}}}}}}T}}$ (where *h* is Planck’s constant, *V* is the hopping integral between the two states, and $\lambda$ is the reorganization energy), we carry out the invert hopping rates (1/${k}_{{{{{{\rm{et}}}}}}}$) as a function of *T* (Fig. 3b), which shows similar trends as the curves from experimental measurement (Supplementary Fig. 14).

The potentiation and depression characteristics of Ag/AgO_*x*_//CAAAAKAAAAK//GaO_x_/EGaIn synapse are subsequently simulated by a continuum charge transport model combining the Nernst–Planck diffusion and Poisson’s equations (NPN)^48,49^. In this model (see “Methods” section for details), both injected Ag^+^ and electrons can migrate and/or diffuse in response to the local electrical field and concentration gradients. Under a constant negative voltage, the continuous injection of Ag^+^ leads to the gradual increase of current (conductance or weight, Fig. 3c). On the reversal of voltage polarity, the current has gradually decreased. This characteristic is very similar to the potentiation/depression of molecular synapse (Fig. 1c–e and Supplementary Fig. 9). In the absence of Ag^+^, however, no current change is measured (Fig. 3c, *black* circle). As the increase of voltage, the concentration of injected Ag^+^ within the peptide junction has also increased, which effectively increases the electron hopping rate and enhances the synaptic potentiation behavior (Fig. 3d). Importantly, the model qualitatively reproduces the double exponential decay of depression current (Fig. 3e vs. 2d and Supplementary Fig. 16 vs. Supplementary Fig. 17). In addition, the *τ*_1_ corresponding to the weak binding mode (chemical gating by free Ag^+^) has gradually increased (note that both the increase of voltage and pulse number lead to more Ag^+^ injection into junction) whereas the *τ*_2_ associated with the stronger binding interaction (coordination effect) is almost not changed (Fig. 3f vs. 2g).

### Emulating synaptic functions

**Fig. 4:**
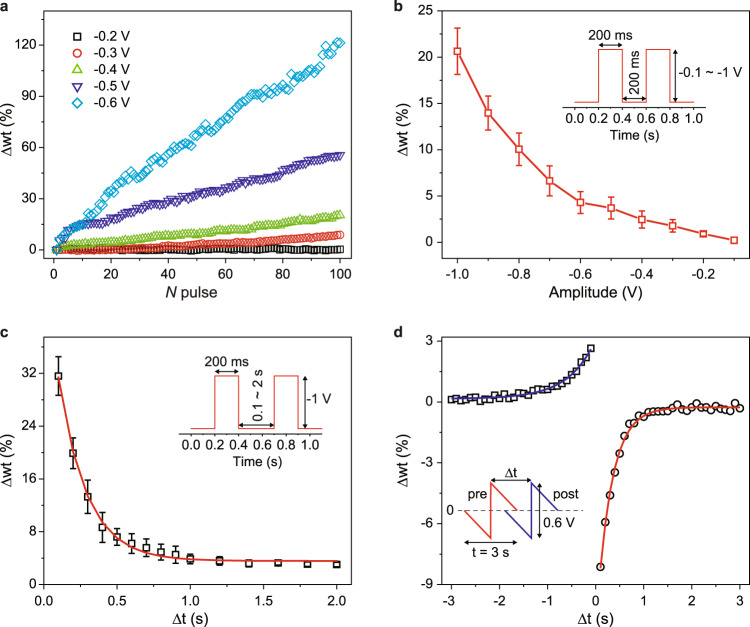
Synaptic plasticity characterization. **a** Synaptic weight change (∆wt) as a function of the number of potentiation pulses under different pulse amplitudes. The pulse width and time interval are 200 ms. Paired pulse behavior of molecular synapse by changing the pulse amplitude **b** (time interval, 200 ms), and time interval **c** (amplitude, −1 V). The width of the pulse is 200 ms. The red line in (**b**) is a guideline of the dependent tendency while the red line in (**c**) shows exponential decay (time constant, 192 ms) with the time interval of the paired-pulse. **d** Spike-timing-dependent plasticity of the molecular synapse. The solid lines are exponential fitting with the time constants of 560 ms (*blue*) and 308 ms (*red*). The insets in (**b**)–(**d**) are the placed pulses. The error bars in (**b**) and (**c**) are based on ten independent devices.

The synaptic plasticity of molecular junction devices is subsequently studied. Figure 4a shows the weight change (∆wt) dependence on the amplitude of pulses. The ∆wt is defined as $\triangle {{{{{\rm{wt}}}}}}=({I}_{n}-{I}_{1})/{I}_{1}$, where *I*_1_ and *I*_*n*_ are the peak currents under the first and *N*th pulses. When the pulse amplitude is lower than −0.3 V (e.g., −0.2 V, *black* curve in Fig. 4a), no apparent conductance change is found, which is consistent with the CV measurement results (Supplementary Fig. 6). Using a pulse with an amplitude beyond −0.3 V could potentiate the molecular synapse and a higher pulse amplitude leads to a steeper increase of weight change, indicating a non-linear modulating characteristic. Then, the weight change in response to the paired pulses is measured by changing the pulse amplitude from −0.1 to −1 V (Fig. 4b, inset) and time interval (∆*t*) from 0.1 to 2 s (Fig. 4c, inset). Similarly, the ∆wt is calculated by using the equation $\triangle {{{{{\rm{wt}}}}}}=({I}_{2}-{I}_{1})/{I}_{1}$ in which *I*_1_ and *I*_2_ are the peak currents of the first and second pulses. The positive ∆wt indicates the paired pulses facilitation (PPF) in the molecular junction, which is used to characterize the temporal or short-term synaptic plasticity^50^. As the increase of paired pulses amplitude, the weight change has dramatically decreased (Fig. 4b). In addition, an exponential decay of ∆wt upon the increase of interval time between two pulses is found (Fig. 4c), which is consistent with bio-synapse behavior.

Next, we switch to emulating the spike-timing-dependent plasticity (STDP), which is classified as long-term plasticity and forms the essential rule of learning and memory law in a biological neural system^51^. The molecular synapse could either be potentiated or depressed according to the time intervals (∆*t*) between presynaptic spikes and postsynaptic spikes, specifically, ∆*t* > 0, depression, ∆*t* < 0, potentiation (Fig. 4d). Again, ∆wt is defined as $\triangle {{{{{\rm{wt}}}}}}=({I}_{t}-{I}_{0})/{I}_{0}$, where *I*_0_ and *I*_t_ are the currents measured 10 s before and after the spikes (under 0.15 V). A longer time interval leads to weaker potentiation and depression. ∆wt could be exponentially fitted (Fig. 4d, *red* and *blue* lines) in which the calculated time constants for pulse intervals at ∆*t* > 0 and ∆*t* < 0 are 529 and 350 ms, respectively. Notably, we measure the weight change at 10 s before and after the spikes, which is far away from the fast relaxation time *τ*_1_ caused by the chemical gating effect (usually below 1 s for consecutive two spikes, see below). The emulation of STDP in molecular synapses should be attributed to the coordination effect with longer relaxation time *τ*_2_.

### Molecular synapse for waveform recognition

**Fig. 5:**
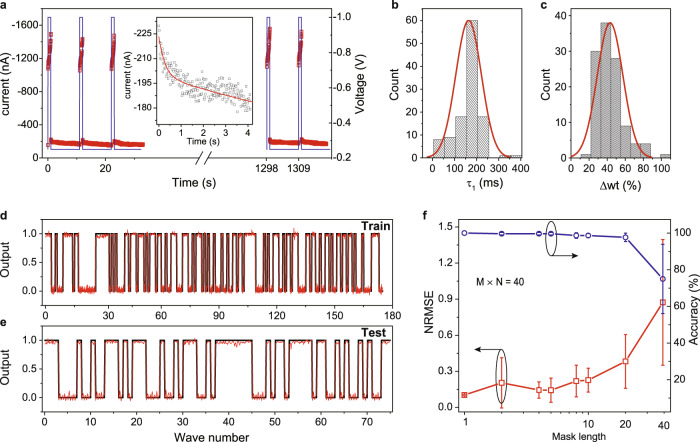
Signal processing based on the molecular synapse. **a** The current response (*red* markers) by placing 120 periodic pulses (*blue* line). The amplitude and width of the write (potentiation) pulse are −1.0 V and 1 s while the amplitude and width of the read (depression) pulse are −0.25 V and 10 s. The inset shows the magnified current decay of the last read pulse, which is fitted by using a double exponential function. **b**, **c** Statistical results of time constant *τ*_1_ (read pulse) and synaptic weight change ∆wt (write pulse) in (a). The solid red lines are fitting to the Gaussian distribution. The performance of reservoir computing system on training **d**, and testing dataset **e** (ground truth, *black* line, output, *red* line). Training and testing data consist of 250 sine and square waveforms. The recognition result is dependent on the output 1 (sine) or 0 (square). **f** The NRMSE (*red* curve) and accuracy (*blue* curve) as a function of mask length. The error bars are based on ten devices.

To perform waveform recognition, the reproducibility and durability of molecular synapses are firstly evaluated. A sequence of alternating write pulses (amplitude, −1.0 V, width, 1 s) and read pulses (amplitude, −0.25 V, width of 10 s) is placed on the molecular synapse (Fig. 5a). The measured current increases quickly with the write pulses while decays under the read pulses (Fig. 5a, inset). No performance degradation has been found for 120 write/read cycles. The time constant *τ*_1_ (by fitting the decay curves) and weight change (calculated from write pulse) are summarized in Fig. 5b, c. The distributions follow the Gaussian law in which the average values are approximately 40% and 150 ms, respectively. The fading characteristics of molecular synapses enable us to mimic the neural network with recursive connections. Combining both *I*–*V* nonlinearity and short-term memory, the molecular synapse or dynamic memristor is then used to recognize waveforms based on the reservoir computing concept^52,53^.

The schematic diagram of a dynamic memristor-based RC system is shown in Supplementary Fig. 18. A simple waveform classification task is used to test the temporal signal processing capability of our RC system. In this task, the input sequence is a random combination of sine and square waveforms, while the desired output is the binary sequence that consists of 0 and 1, representing sine and square waveforms respectively. The system recognizes sine and square waveforms through 4 steps. In step 1, sampling several points per waveform. The dataset consists of sine and square waveforms are divided into train and test datasets. For example, we sample 8 points per waveform and all of the sampling points from one dataset are arranged as a vector, termed sampling vector. In step 2, transform the sampling vector into a voltage vector through a mask. The mask is a randomly generated vector consisting of 1 and −1 with a length of mask length (ML). Each sampling point is expanded to ML points by multiplying by mask. The length of the sampling vector then becomes ML times, termed voltage vector. Taking a mask length of 2, for example, there are four possibilities of the mask according to permutation and combination. For a given RC unit, all of the input data multiply by the same randomly selected mask. For a given parallel RC system, each RC unit selects a mask randomly. In step 3, place voltage pulses to RC units and collect state information. Voltage pulses with different amplitudes and the same pulse width are generated according to the voltage vector. Currents measured at high voltage reflect states of RC units during classification, termed current vector. In step 4, calculating output categories by the linear combination of the current vector from all RC units. In the training process, the weights of the linear combination are trained by linear regression with a training dataset. In the testing process, the trained weights are used to infer classification results with the test dataset.

Figure 5d, e shows the prediction results on the training dataset and test dataset. The performance on the training dataset reflects whether the amount of dataset is enough while the test dataset shows the capability of classification. The normalized root mean square error (NRMSE) is used to evaluate the performance of the RC system on the test dataset, which is described as ${{{{{\rm{NRMSE}}}}}}=\sqrt{\frac{\left\langle {{||y}\left(t\right)-{y}_{{{{{{\rm{target}}}}}}}\left(t\right){||}}^{2}\right\rangle }{\left\langle {{||y}\left(t\right)-{y}_{{{{{{\rm{target}}}}}}}\left(t\right){||}}^{2}\right\rangle }}$ in which y(t) is the prediction of RC system, *y*_target_(*t*) is the ground truth, ||•|| denotes the Euclidean norm, and <•> denotes the empirical mean. The influence of mask length on NRMSE is shown in Fig. 5f. Considering that the mask process and parallel RC units expand the amount of input data, we keep the size of the reservoir fixed (*M* × *N* = 40), *M*, mask length, *N*, number of memristors) during comparison. RC system achieves lower NRMSE and smaller variation when the mask length is smaller (Fig. 5f, *red* curve). The recognition result could be regarded as sine or square when the system output is closer to 1 or 0. Comparing the recognition results to ground truth, our RC system achieves 100% accuracy when the mask length is small (Fig, 5f, *blue* curve).

In conclusion, we have demonstrated a molecular synapse in which the conductance or synaptic weight could be gradually modulated. Different from previous digital switching in molecular RRAM, the dynamic current response enables us to emulate the typical synaptic plasticity and perform waveform recognition based on the reservoir computing concept. In addition, we propose that the electric field induces Ag^+^ injection into peptide monolayer where the interaction between Ag^+^ and functional groups accounts for the dynamic conductance modulation and the short-term (chemical gating) and long-term (coordination) synaptic behaviors. Using individual molecule or SAM as components is probably the ultimate goal of silicon electronics. Similarly, emulating the biological synapses, neurons, and even neural networks within the same length scale is also worth the effort due to the rewards of energy and integration density upon device miniaturization. Looking forward, we expect our preliminary demonstration could inspire more work not only on the fundamental bio-devices but also on the hardware implementation of complex tasks by using single or monolayer molecules. Importantly, these electronic devices are made up of biomolecules that could be expected to directly interface with networks to monitor, interfere with, and even direct biosignals.

## Methods

### Materials

The peptides with a purity of over 95% are purchased from Bank peptide biological technology, China. Dodecanethiol is purchased from Aladdin Chemistry and used without further purification. HS-PEG8-CH_2_CH_2_COOH with a purity of over 95% is obtained from Sigma-Aldrich. Ag source for evaporation is obtained from CNM Materials (China New Metal Materials).

### Device fabrication

The molecular synapse device contains a bottom Ag/AgO_*x*_ electrode, peptide SAM, and a liquid GaO_*x*_/EGaIn top electrode, Ag/AgO_*x*_//CAAAAKAAAAK//GaOx/EGaIn.

The bottom Ag/AgO_*x*_ electrode is prepared by a template stripping method^54^. Typically, a 200 nm-thick Ag film is firstly deposited on a Si (100) wafer by electron beam evaporation method under a rate of 0.4 Å/s. The Ag film is subsequently thermally annealed on a hot plate at 150 °C for 40 min in the air with RH around 60%. Epoxy (~100 μL, EPO-TEK 353ND) is then pipetted onto the Ag/AgO_*x*_ film, followed by degassing in a vacuum chamber (~0.1 mbar) for 15 mins. At the same time, a glass slide (${2*3\,{{{{{\rm{cm}}}}}}}^{2}$) is precleaned in 2 M NaOH alkaline solutions (isopropyl alcohol/H_2_O, 2:1, v/v) for 24 h, washed with DI water and dried in a flowing nitrogen gas. The cleaned glass slide is then placed onto the epoxy/Ag/AgO_*x*_. After carefully squeezing out redundant epoxy, the slide is subsequently cured at 60 °C for 12 h in an oven. Finally, the Ag/AgO_*x*_ electrode is lifted off from the silicon wafer by using a razor blade.

To assemble the peptide (CAAAAKAAAAK, C, A, and K are cysteine, alanine, and lysine) monolayer on Ag/AgO_*x*_ electrode, the lifted-off slide is immediately immersed in 0.5 mM ethanolic solutions of the peptide for 12 h^55^. The Ag/AgO_*x*_//peptide is then carefully rinsed in ethanol three times to remove extra peptides and gently dried using flowing nitrogen gas. SAMs of peptide C(GABA)(GABA)D(GABA)(GABA)D (C, (GABA), and D are cysteine, γ-aminobutyric acid, and aspartic acid) are assembled in a 0.5 mM water solution. Before assembling, the dissolved O_2_ in the solution is removed by purging N_2_ for 3 h. The lifted-off Ag/AgO_*x*_ electrode is immediately immersed in the solution of the peptide for 12 h in a glove box free of O_2_. The Ag/AgO_*x*_//peptide is then rinsed in deionized (DI) water three times and gently dried with nitrogen gas. The Ag/AgO_*x*_//HS-PEG8-CH_2_CH_2_COOH SAM is prepared with a similar procedure to the Ag/AgO_*x*_//CAAAAKAAAAK SAM except for the ethanolic solution. To prepare Ag/AgO_*x*_//dodecanethiol SAM, 0.5 mM dodecanethiol toluene solution is used for the assembling of the monolayer, followed by washing with toluene and drying with flowing nitrogen gas.

To prepare Ag//peptide and Ag//peptide-M^*n*+^ SAMs (M^*n*+^ is the metal cation), Ag electrodes are prepared by the same template stripping method, however, the Ag films are not thermally annealed. The binding of M^*n*+^ is performed by immersing Ag//peptide SAMs in the nitrate solutions for 15 min. The Ag//peptide-M^*n*+^ SAMs are then carefully rinsed in DI water three times to remove extra metal cations and gently dried using flowing nitrogen gas. 20, 40, 60, 80, and 100 mM AgNO_3_, NaNO_3_ and Ca(NO_3_)_2_ are used for the control experiments (Supplementary Fig. 13). 10 mM AgNO_3_ solution is used for preparing Ag//CAAAAKAAAAK-Ag^+^//GaO_*x*_/EGaIn devices for the temperature-dependent electrical test (Supplementary Fig. 14).

The liquid GaO_*x*_/EGaIn top electrode is fabricated according to the previous report^11^. Briefly, two molds (one bottom channel and one top channel molds) are required to prepare PDMS blocks. Both molds are prepared by photolithography. The two PDMS blocks are carefully aligned to ensure a through-hole connection for liquid metals. To fill the PDMS channel with liquid metals, a two-step pumping method is used. There are five through-holes in one PDMS slab, thus five molecular junctions can be fabricated by landing one PDMS slab on Ag/AgO_*x*_//peptide SAM. The active area of one molecular junction is *π* ∗ (10 μm)^2^. In addition, new devices can be fabricated by stripping the PDMS slab and landing it on the new place of the bottom SAM. Usually, a PDMS slab can be used to prepare dozens of molecular junctions before failure.

### Atomic force microscopy (AFM) characterization

The AFM characterization is performed on the Bruker Multimode 8-HR instrument in tapping mode. To minimize the impact of the tip on the surfaces, soft AFM cantilevers with a force constant of around 0.4 N/m are used for the characterization.

The local roughness of the surfaces is analyzed according to a reported method^56^. Briefly, AFM images are first converted to black and white photographs in the NanoScope Analysis software, in which the RGB value of each pixel could be easily mapped to the height value of the corresponding area by a python script. Each AFM photograph is then imported into the script as a matrix of 4000 × 4000 RGB elements. The RGB matrix is subsequently transferred to a new matrix containing actual height information of the scanned area (1 μm × 1 μm) according to the color scale bar and is equally divided into 100 small sub-matrices. The RMS roughness of each local scanned area (100 nm × 100 nm) is calculated according to ${{{{{\rm{RMS}}}}}}=\,\sqrt{\frac{1}{n}\sum {(Z-{Z}_{{{{{{\rm{mean}}}}}}})}^{2}}$, where *Z* is the height value of each element in a sub-matrix and *n* is the element number of the sub-matrix. Each distribution of local roughness of different surfaces is calculated from 6 scanned areas of three different samples.

To prepare a sample for measuring the CAAAAKAAAAK SAMs thickness, a freshly prepared PDMS block is put on part of the lifted-off Ag/AgO_*x*_ electrode. Next, the remaining electrode surface is modified with peptide molecules following the same procedure as Ag/AgO_*x*_//peptide electrodes. Since there is no infiltration of solution into the PDMS region, a clear boundary is obtained after peeling off the PDMS, which is subsequently recorded by AFM.

### Angle-resolved X-ray photoelectron spectroscopy

The physicochemical properties of both Ag/AgO_*x*_ electrode and peptide SAMs are characterized by using Angle-resolved X-ray photoelectron spectroscopy (AR-XPS, Thermo Scientific™ Nexsa™ XPS). The samples are prepared in the cleanroom and stored in a box filled with N_2_ before being loaded into the AR-XPS chamber. The AR-XPS measurements are performed under an ultra-high vacuum with a pressure of 5 × 10^−9^ mbar. The photon energy is calibrated relative to the adventitious carbon C *1s* peak (284.8 eV). The angle between the analyzer and the incident beam is fixed at 58°. Spectra of the SAMs are recorded with the take-off angles (θ, the angle between the analyzer and the substrate surface) *θ*_1_ = 90° and *θ*_2_ = 40°, while the spectra of the Ag/AgO_*x*_ electrode are measured at *θ* = 40°. The spectra are fitted to the Voigt functions (ratio of Lorentzian/Gaussian, 3/7) with a Shirley background correction.

Based on the AR-XPS results, the thickness *d*_SAM_ and the surface coverage Ψ_SAM_ of the peptide SAMs are calculated by using a procedure according to previous reports^57–59^. Briefly, the thickness *d*_SAM_ is obtained from the S *2p* spectra, and calculated according to the equation$\,\,{d}_{{{{{{\rm{SAM}}}}}}}=\frac{{{{{{\rm{\lambda }}}}}}{{{{{\rm{sin }}}}}}{\theta }_{1}{{{{{\rm{sin }}}}}} {\theta }_{2}\left\{{{{{{\rm{ln}}}}}}\left(\frac{{I}_{{\theta }_{1}}}{{I}_{{\theta }_{2}}}\right)+{{{{{\rm{ln}}}}}}\left[1-{{{{{\rm{e}}}}}}^{-\frac{x}{\lambda {{{{{\rm{sin }}}}}} {\theta }_{2}}}\right]-{{{{{\rm{ln}}}}}}\left[1-{{{{{\rm{e}}}}}}^{-\frac{x}{\lambda {{{{{\rm{sin }}}}}} {\theta }_{1}}}\right]\right\}}{{{{{{\rm{sin }}}}}}{\theta }_{1}-{{{{{\rm{sin }}}}}} {\theta }_{2}}\,+{d}_{{{{{{\rm{Ag}}}}}}-{{{{{\rm{S}}}}}}}$, where λ (～11 Å)^60^ is the inelastic mean free path of photoelectrons with kinetic energy of ～180 eV in peptide SAMs, *θ* is the take-off angle, *x* (~1.5 Å) is estimated by the length of S–C bond, *d*_Ag–S_ (~1.8 Å) is estimated from the length of Ag–S bond, and the effective intensities *I*_θ_ are calculated according to ${I}_{\theta }=I\cos ({90}^{o}-\,\gamma )$ (where *I* is the intensity from the spectra and *γ* is the angle of the incident beam and the substrate). The surface coverage Ψ_SAM_ is determined by the Ag *3d* spectra of the peptide and dodecanethiol SAMs. It is calculated according to the equation $\frac{{I}_{0,{{{{{\rm{Ag}}}}}}-{{{{{\rm{S}}}}}}}\left({{{{{\rm{peptide}}}}}}\right)}{{I}_{0,{{{{{\rm{Ag}}}}}}-{{{{{\rm{S}}}}}}}\left({{{{{\rm{S{C}}}}}}}_{12}{{{\rm{H}}}}\right)}=\,\frac{{\Psi }_{0,{{{{{\rm{Ag}}}}}}-{{{{{\rm{S}}}}}}}\left({{{{{\rm{peptide}}}}}}\right)}{{\Psi }_{0,{{{{{\rm{Ag}}}}}}-{{{{{\rm{S}}}}}}}\left({{{{{\rm{S{C}}}}}}}_{12}{{{\rm{H}}}}\right)}$, where ψ_0, Ag–__S_ (SC_12_H) is assumed to the theoretical value 6.6 × 10^14^ /cm^2^ and the Ag *3d* intensity attenuated by the sulfur atom *I*_0, Ag–__S_ is calculated by ${I}_{0,{{{{{\rm{Ag}}}}}}-{{{{{\rm{S}}}}}}}={I}_{{{{{{\rm{Ag}}}}}}}/{{{{{\rm{e}}}}}}^{-d/{\lambda }_{{{{{{\rm{Ag}}}}}}}}$ (where *d* is the distance from the sulfur to the top surface of the film and *λ*_Ag_ ~17 Å^60^ is the inelastic mean free path of photoelectrons with kinetic energy ~368 eV in organic SAMs). To calculate the surface coverage of the peptide, we assume the surface of the lifted-off Ag/AgO_*x*_ has a structure close to an idea Ag surface (as indicated in Supplementary Fig. 5) and the photoelectrons with high energy behave similarly in organic SAMs^60^.

### Ellipsometry characterization

Alternatively, the thickness of the peptide SAMs is measured by an auto ellipsometer (SENTECH SE 850 DUV). The spectral range is 190–1000 nm and the incident angle is 70°. The optical model for the automatic calculation by the equipped software consists of two layers, i.e., the substrate and the bulk layers. The optical constants (refractive index and extinction coefficient) of the Ag substrate and peptide layer are assigned according to the Brendel oscillator model and the Tauc–Lorentz model respectively. The freshly prepared samples are immediately loaded onto the sample stage. The averaged thickness is obtained from three different spots on three independent Ag/AgO_*x*_//CAAAAKAAAAK samples.

### Electrical measurement

The pulse/voltage is placed on the top liquid electrode which is wired out by using a Pt wire immersed in the GaO_*x*_/EGaIn liquid in the inlet of the PDMS slab. The bottom Ag or Ag/AgO_*x*_ electrode is ground. The electrical characteristics are measured by using a Keysight B2912A electrometer. The signal processing is carried out with a homemade test system based on a DAQ card (NI PCIe-6361) and an SR570 Low-Noise Current Preamplifier. The cyclic voltammetry is recorded on the electrochemical workstation CHI660E.

### Theory details

The coupling transport of ionic and electronic charges within the peptides is modeled by the coupled PNP equations. The PNP model is based on both the Nernst–Planck theory describing the diffusion of ions and electrons, and the Poisson theory describing the electrostatic potential. For a system geometry approximated as one-dimensional ($\Omega=\left[0,L\right]$, where *L* is the distance between electrodes), the transport of charged species *i* (Ag^+^ and electrons) through the peptides is modeled by the one-dimensional PNP equations as

1 $$\frac{\partial {C}_{n}}{\partial t}=\frac{\partial }{\partial x}\left({{D}_{n}\frac{\partial {C}_{n}}{\partial x}+{z}_{n}e{D}_{n}\beta {C}_{n}\frac{\partial \phi }{\partial x}}\right)_{,}$$

2 $$\frac{\partial {C}_{e}}{\partial t}=\frac{\partial }{\partial x}\left({{D}_{e}\frac{\partial {C}_{e}}{\partial x}+{z}_{e}e{D}_{e}\beta {C}_{e}\frac{\partial \phi }{\partial x}}\right)_{,}$$

3 $$-\frac{{\partial }^{2}\phi }{\partial {x}^{2}}=\frac{e}{{\varepsilon }_{0}\varepsilon }({z}_{e}{C}_{e}+{z}_{n}{C}_{n})$$

where ${C}_{n},{C}_{e}$ is the concentration of the Ag^+^ and electrons; ${D}_{n},{D}_{e}$ is the spatial-dependent diffusion coefficient, $\phi$ is the electrostatic potential, and the constant $\beta=1/{k}_{B}T$ is the inverse Boltzmann energy where ${k}_{B}$ is the Boltzmann constant and *T* is the absolute temperature. We assume that the dielectric permittivity is constant in the whole peptide. ${\varepsilon }_{0}$ is the vacuum dielectric constant and the relative dielectric constant $\varepsilon$ is set as 4.0. There is no flux boundary surrounding the EGaIn electrode, which prevents Ag^+^ from penetrating through the EGaIn electrode, i.e., the ion flux is zero at *x* = *L*.

To estimate the required parameters from the model, we consider the relevant experimental information. First, the experimental time is about several seconds, therefore the diffusion coefficient of Ag^+^ can be approximated by $\left\langle {x}^{2}\right\rangle=2{Dt}$, in which experimentally, *L* = 4 nm and the diffusion coefficient is estimated to be ${D}_{n} \sim 1\times \frac{{10}^{-18}{m}^{2}}{s}$. According to the transmission probability of electrons shown in Fig. 3b, the Marcus hopping rate of electrons with Ag^+^ binding is about 30 times higher than that of electrons without Ag^+^ binding. In addition, we believe that during the process of potentiation, as the injection of Ag^+^ into peptide monolayer, the transmission probability of electrons increases continuously until a maximum value is reached as time evolves; however, the transmission probability of electrons decreases with time during the process of depression. Based on these information and experimental results, we design a function for the diffusion coefficient of electrons as follow

$${D}_{e}\left({{{{{\rm{t}}}}}}\right)=\left\{\begin{array}{cc}{{{{{\rm{A}}}}}}{{e}}^{{at}},\hfill & 0 \, < \, {{{{{\rm{t}}}}}} \, < \,{t}_{p} \hfill\\ {{{{{\rm{B}}}}}}{{e}}^{b\left(t-{t}_{p}\right)},\hfill & \, {t}_{p} \, < \, {{{{{\rm{t}}}}}} \, < \, \,{t}_{p}+0.1{t}_{d}\\ {{{{{\rm{B}}}}}}{{{e}}^{0.1b}{{e}}}^{c\left(t-{t}_{p}-0.1{t}_{d}\right)},& \,{t}_{p}+0.1{t}_{d} \, < \,{{{{{\rm{t}}}}}} \, < \,{t}_{d}\end{array}\right.$$

where ${t}_{p}$, ${t}_{d}$ represent the process of potentiation and depression. To fit with the experimental data, we set $A=1\times {10}^{-17}$, $B=3\times {10}^{-16}$, *a* = 6.8, *b* = −7.4, *c* = −3.4 in our simulation.

The coupling Eqs. (1–3) are then solved by using a commercial finite element solver (COMSOL 5.4). When a negative voltage is placed on GaO_*x*_/EGaIn electrode, the current increases exponentially as the injection of Ag^+^ into the peptide enhances the electrons hopping transmission probability. During the process of depression, as a positive voltage is placed on the GaO_*x*_/EGaIn electrode, thus Ag^+^ cations unbind with functional groups on peptide and lead to a decrease of the current with time.

Since the long carbo–nitrogen back-bone is saturated, the peptide holds a quite wide HOMO-LUMO gap, leading to an extremely big transmission interval (bigger than 10 eV). After Ag^+^ injection, extra peaks appear in the transmission gap due to Ag^+^ introducing new transmission channels.

## Supplementary information

Supplementary Information

## Data Availability

The data that support the findings of this study are available from the corresponding authors upon reasonable request.

## Code availability

The code used in this study is available from the corresponding authors upon reasonable request.
