# Supplementary material for: An artificial synapse based on molecular junctions
Source: Nat Commun. 2023 Jan 16;14:247. doi: 10.1038/s41467-023-35817-5 (PMC9842743; doi:10.1038/s41467-023-35817-5)
Supplement: Supplementary file 1 — Supplementary Information [file 41467_2023_35817_MOESM1_ESM.pdf]

Supplementary Information

An artificial synapse based on molecular junctions

Yuchun Zhang[1†], Lin Liu[1,2†], Bin Tu[1†], Bin Cui[3†], Jiahui Guo[1,2], Xing Zhao[1], Jingyu Wang[1,2], and Yong Yan[1,2,4*]

[1]*CAS Key Laboratory of Nanosystem and Hierarchical Fabrication, CAS Center for Excellence in Nanoscience, National Center for Nanoscience and Technology, Beijing 100190, China*

[2]*University of Chinese Academy of Sciences, Beijing, 100049, China*

[3]*School of physics, Shandong University, Jinan, 250100, China*

[4]*Department of Chemistry, School of Chemistry and Biological Engineering, University of Science and Technology Beijing, Beijing 100083, China*

[†]*These authors contributed equally to this work*

*Correspondence to: yany@nanoctr.cn (Y.Y.)

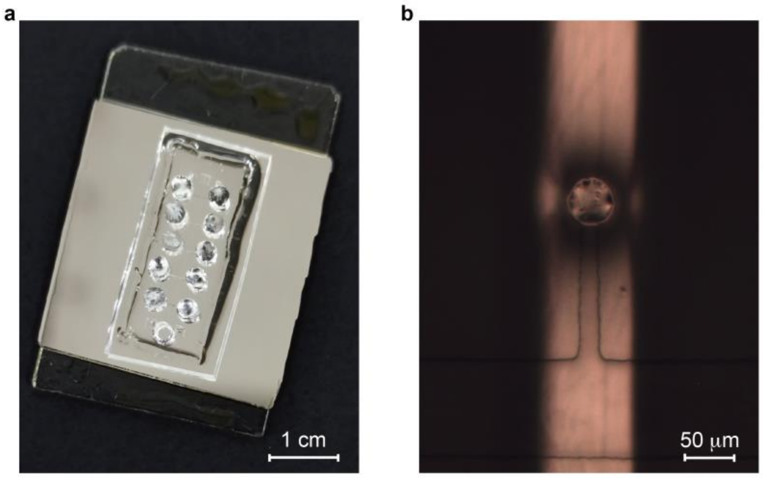

Fig. S1 | Device architecture. a, Photograph of a chip containing five molecular synapses. **b**, Bottom view of a through-hole which is filled by liquid GaO_x/EGaIn.

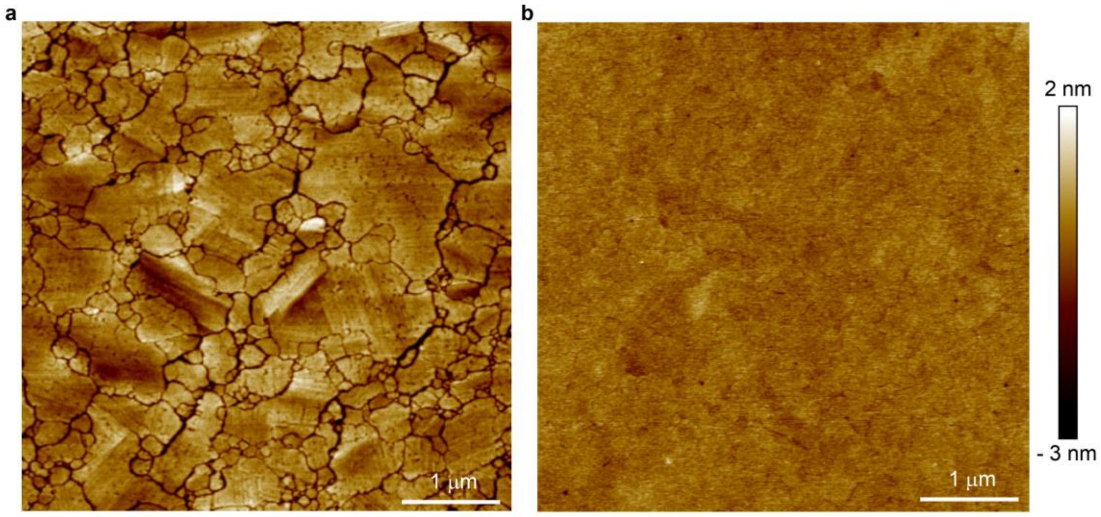

Fig. S2 | AFM images of the lifted-off Ag/AgO_x electrode without (a) and with (b) peptide SAMs. After molecule assembling, the gaps between Ag grains have decreased and a uniform monolayer of peptide molecules is formed on the electrode surface.

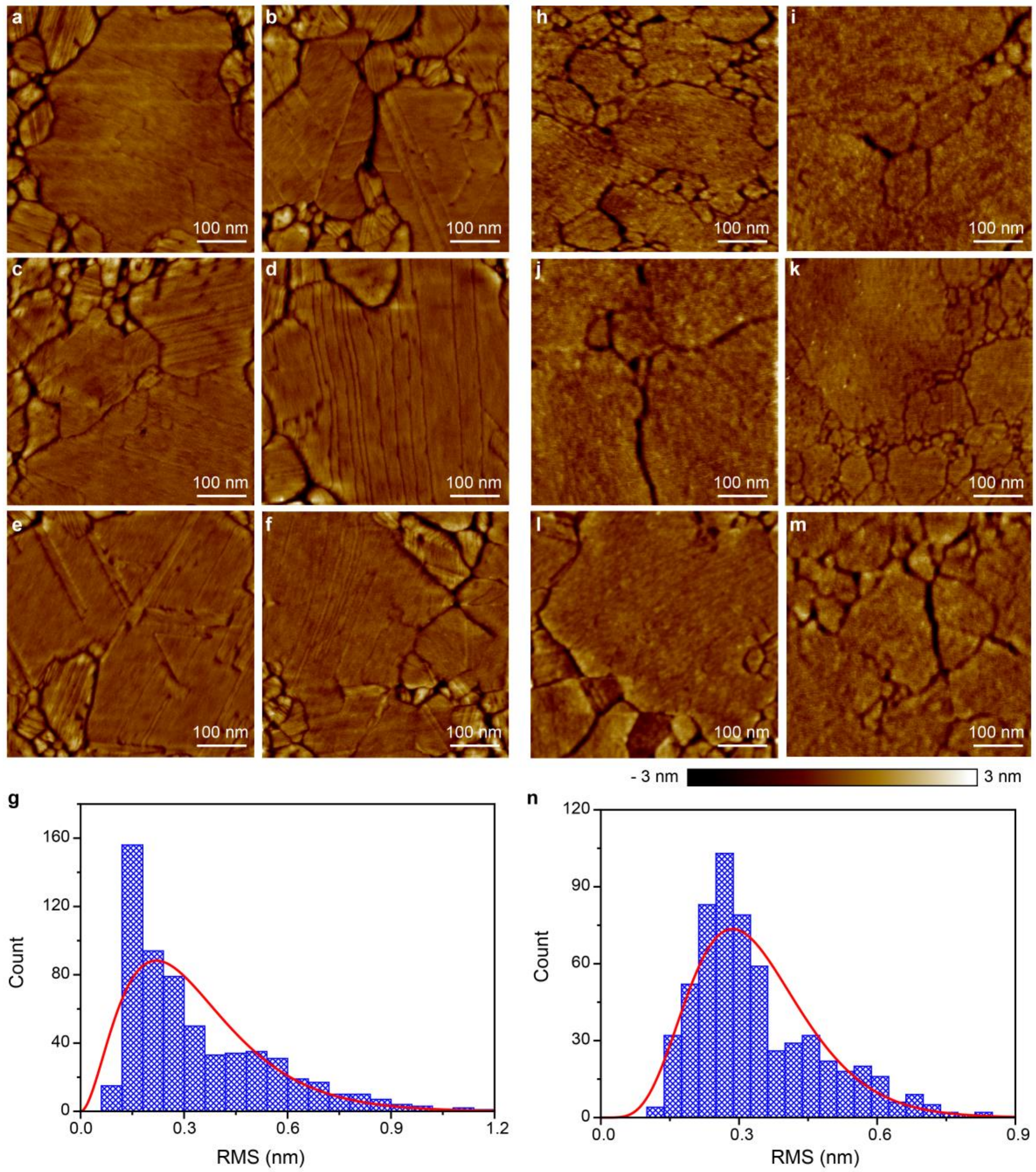

Fig. S3 | Local roughness distributions of the lifted-off Ag/AgO$_x$ electrode without/with peptide SAMs. a-f (**h-m**), The AFM images of three different samples without (with) peptide SAMs. **g** (**n**), The local surface roughness distribution calculated from **a-f** (**h-m**). The solid red lines in (g) and (n) are Gamma fittings to the distributions. The peptide SAMs on the electrodes are densely packed and uniform. Compared to the freshly lifted-off Ag/AgO$_x$ surface, the electrodes with peptide SAMs have a local roughness distribution with the peak upshifted

around 0.1 nm. In addition, the shorter tail of the RMS distribution in the peptide SAMs is found which could be attributed to the shrinkage of Ag grain gaps by molecule assembling at the grain edges.

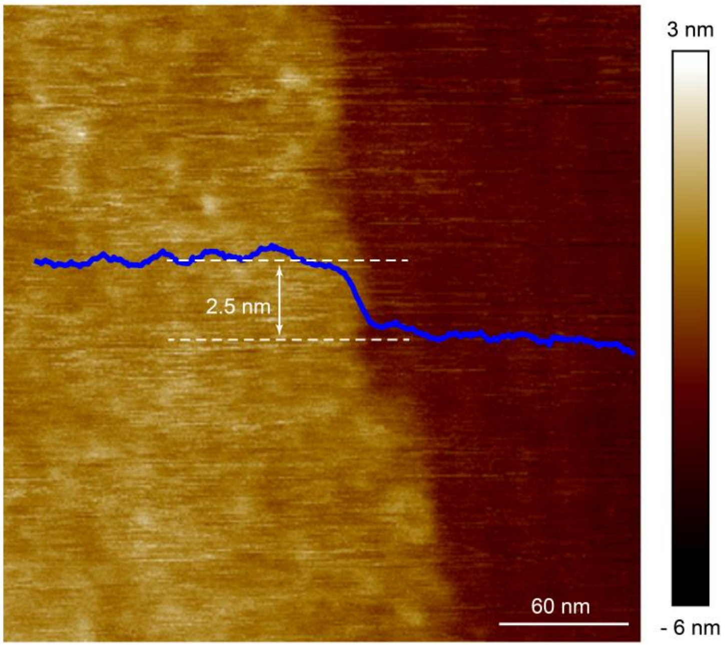

Fig. S4 | Thickness measurement of the CAAAAKAAAAK SAMs on the Ag/AgO$_x$ electrode. The thickness of the molecular layer is around 2.5 nm.

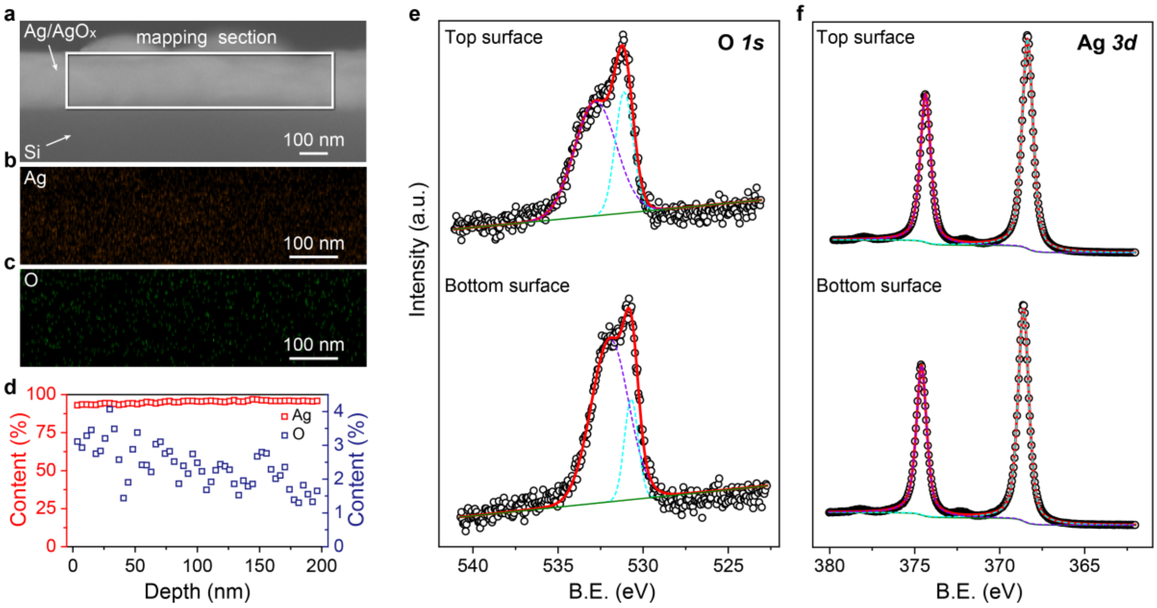

Fig. S5 | Characterization of element distribution in the Ag/AgO$_x$ electrode. a, Cross-section SEM image of a typical Ag/AgO$_x$ film on a silicon substrate. **b** and **c**, Energy-dispersive spectroscopy (EDS) element mapping images of Ag and O. **d**, Corresponding Ag and O elements distributions. **e** and **f**, O *1s* and Ag *3d* XPS spectra of the top and the bottom surfaces of Ag/AgO$_x$ film. The top surface is measured before lift-off while the bottom surface is measured after lift-off. Note: the bottom surface is used to bind peptide molecules.

The EDS element mapping indicates a slow increase of Ag and an apparent decrease of O from the top to the bottom surfaces of the Ag/AgO$_x$ electrode (**Supplementary Fig. 5a-d**). In **Supplementary Fig. 5e**, both O *1s* spectra contain two main components, *i.e.*, B.E. = 532.9 eV and B.E. = 531.2 eV for the top surface, and B.E. = 532.0 eV and B.E. = 531.1 eV for the bottom surface. As Ag-O contributions to the O *1s* peaks can overlap the absorbed oxygen or hydroxyl groups, no definite assignments could be given to the O *1s* peaks[1]. However, the Ag $3d_{5/2}$ peak (**Supplementary Fig. 5f**) of the top surface at 368.3 eV could be associated with Ag-O bonding while the peak at 368.6 eV of the bottom surface should be associated with the

Ag-Ag bonding[2]. Both experiments demonstrate a gradient distribution of AgO_x due to the thermal annealing procedure in air. However, the bottom surface used for the assembling of peptide SAMs is almost intact.

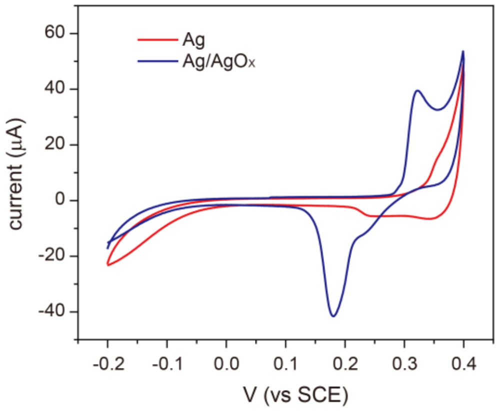

Fig. S6 | Electrochemical Characterization of Ag and Ag/AgO_x electrode. Cyclic voltammetry curves of peptide SAM covalently bonded on Ag (*red*) and Ag/AgO_x (*blue*) electrodes. The measurements are performed in 0.1 M $HClO_4$ electrolytes with a scan rate of 10 mV/s. The reference and counter electrodes are saturated calomel and Pt wire respectively.

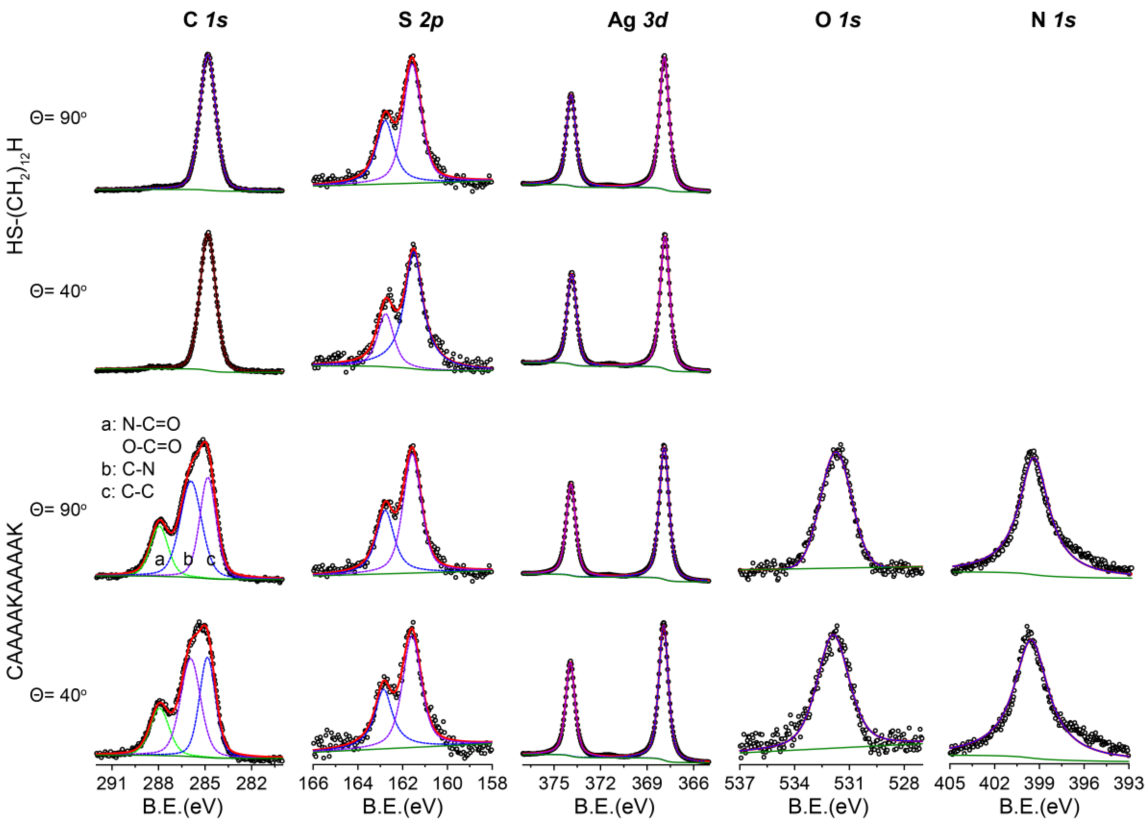

Fig. S7 | AR-XPS Characterization of SAMs on the lifted-off Ag/AgO$_x$ electrodes. Angle-resolved C *1s*, S *2p*, and Ag *3d* spectra of the HS-$(CH_2)_{12}$H SAMs (*top* half) and angle-resolved C *1s*, S *2p*, Ag *3d*, O *1s*, N *1s* spectra of the CAAAAKAAAAK SAMs (*bottom* half).

Based on AR-XPS results, the thickness of the CAAAAKAAAAK SAMs is around 21 Å while the thickness of the HS-$(CH_2)_{12}$H SAMs is around 16 Å. The surface coverage of the peptide SAMs is determined approximately $3.6 \times 10^{14}/cm^2$. Alternatively, the thickness of the peptide SAMs measured by Ellipsometry is around 25 Å. The length of the CAAAAKAAAAK molecule is estimated at 40 Å based on the MD simulations. Therefore, the tilt angle of the peptide molecules in the SAMs could be estimated at around 55°. It could be concluded that CAAAAKAAAAK molecules form a densely-packed structure on the Ag/AgO$_x$ electrode.

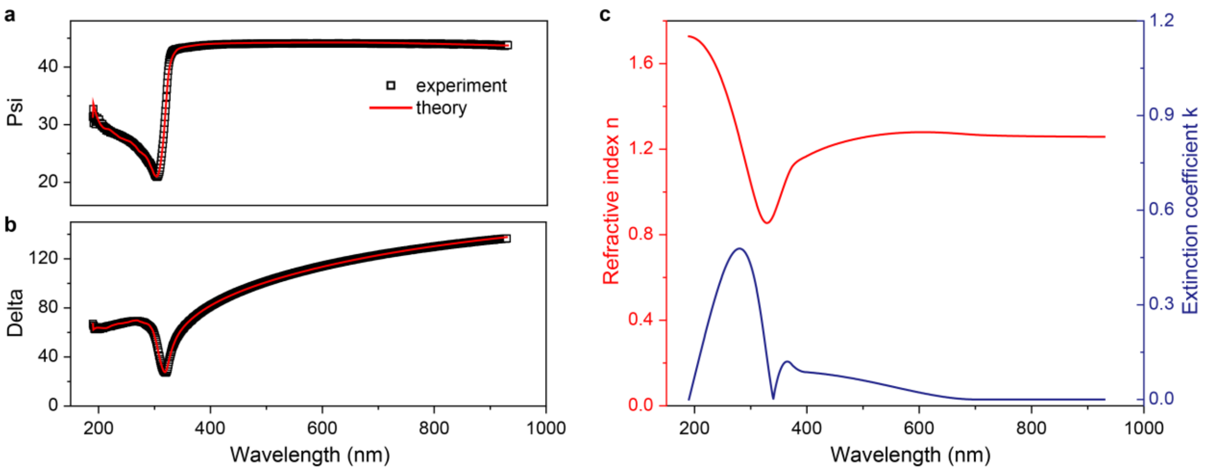

Fig. S8 | Ellipsometry Characterization of SAMs. **a**, Ratio of amplitude change Psi, and **b**, the difference in phase shift Delta obtained from the experimental (*black* squares) and the theoretical calculation by the equipment (*red* lines). **c**, Optical constants of refractive index n (*red* line, *left* y-axis) and extinction coefficient k (*blue* line, *right* y-axis) assigned according to the optical models for the calculations by the equipment. The thickness of the peptide layer is estimated at around 2.5 nm from the measurements.

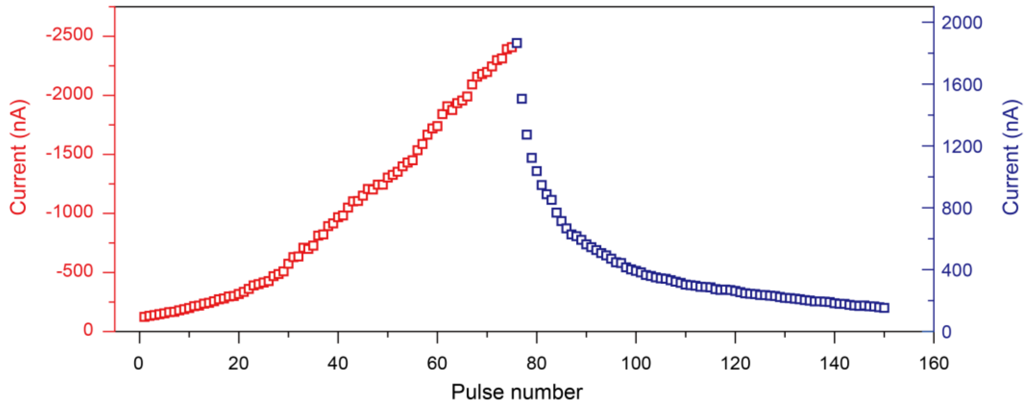

Fig. S9 | Potentiation and depression of molecular synapse by triangle pulses. The current peaks under each triangle pulse (Fig.1c and Fig. 1d) are recorded to plot this curve.

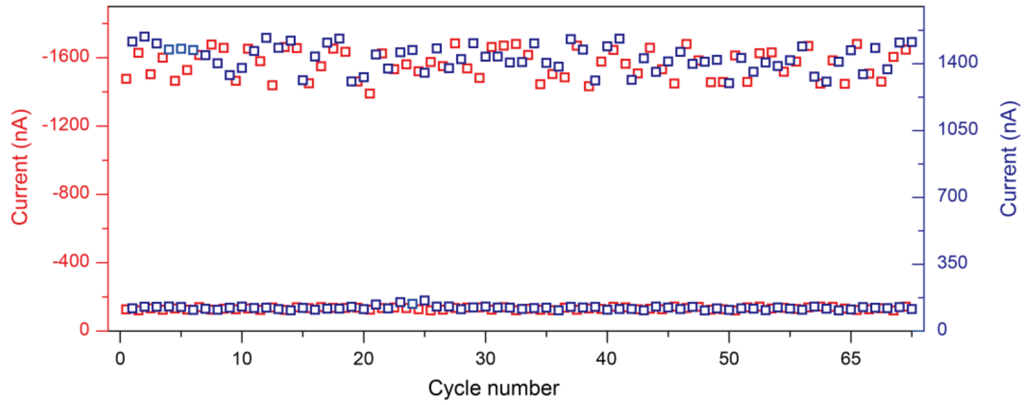

Fig. S10 | Endurance test of the molecular synapse. The current peaks of potentiation and depression pulses are recorded for 65 cycles. For each potentiation and depression process, there are 30 square pulses. The pulse amplitude is ±0.45 V and the width is 0.5 s.

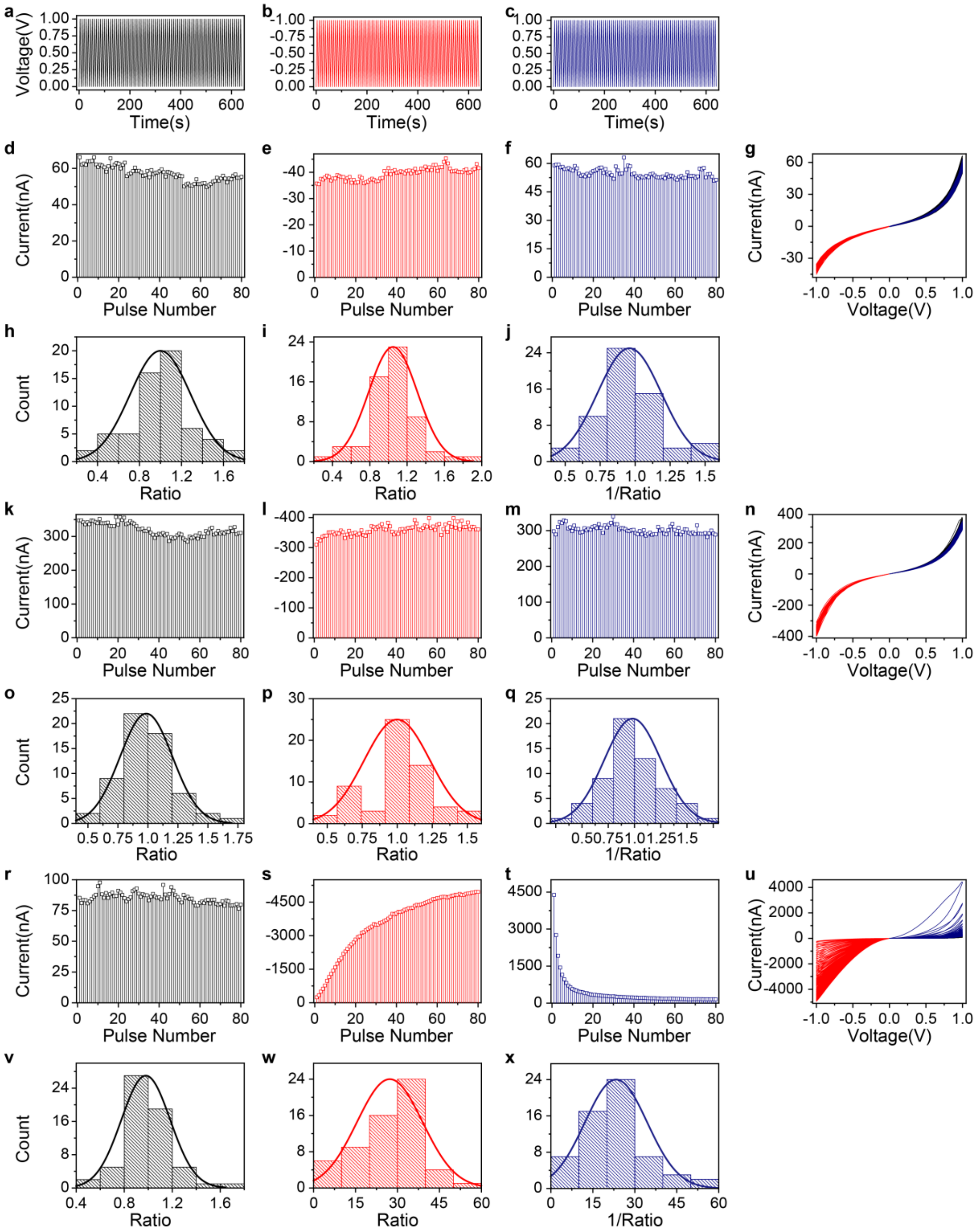

Fig. S11 | Electrical characteristics of the Ag//CAAAAKAAAAK//GaO_x/EGaIn, Ag/AgO_x//HS-$(CH_2)_{12}$H//GaO_x/EGaIn, and Ag/AgO_x//CAAAAKAAAAK//GaO_x/EGaIn devices. Sequences of 80 positive **a**, negative **b**, and positive **c**, triangle pulses (amplitude, 1 V, width, 8 s) are consecutively placed on the junctions. The bottom Ag/AgO_x or Ag electrode

is grounded. **d** to **f** (**k** to **m** and **r** to **t**), The measured current peaks of a typical Ag//CAAAAKAAAAK//GaO_x/EGaIn (Ag/AgO_x//HS-$(CH_2)_{12}$H//GaO_x/EGaIn and Ag/AgO_x//CAAAAKAAAAK//GaO_x/EGaIn) device under sequences of 80 triangle pulses shown in (a) to (c). **g** (**n** and **u**), Corresponding current-voltage characteristics of the Ag//CAAAAKAAAAK//GaO_x/EGaIn (Ag/AgO_x//HS-$(CH_2)_{12}$H//GaO_x/EGaIn and Ag/AgO_x//CAAAAKAAAAK//GaO_x/EGaIn) device. **h** to **j** (**o** to **q** and **v** to **x**)**,** The histograms with a Gaussian fit of current change ratio of 60 Ag//CAAAAKAAAAK//GaO_x/EGaIn (Ag/AgO_x//HS-$(CH_2)_{12}$H//GaO_x/EGaIn and Ag/AgO_x//CAAAAKAAAAK//GaO_x/EGaIn) devices under sequences of 80 triangle pulses shown in (a) to (c). The current change ratio is calculated according to Ratio = I_{80}/I_1, where I_{80} (I_1) is the measured current of the 80th (1st) pulse shown in (a) to (c).

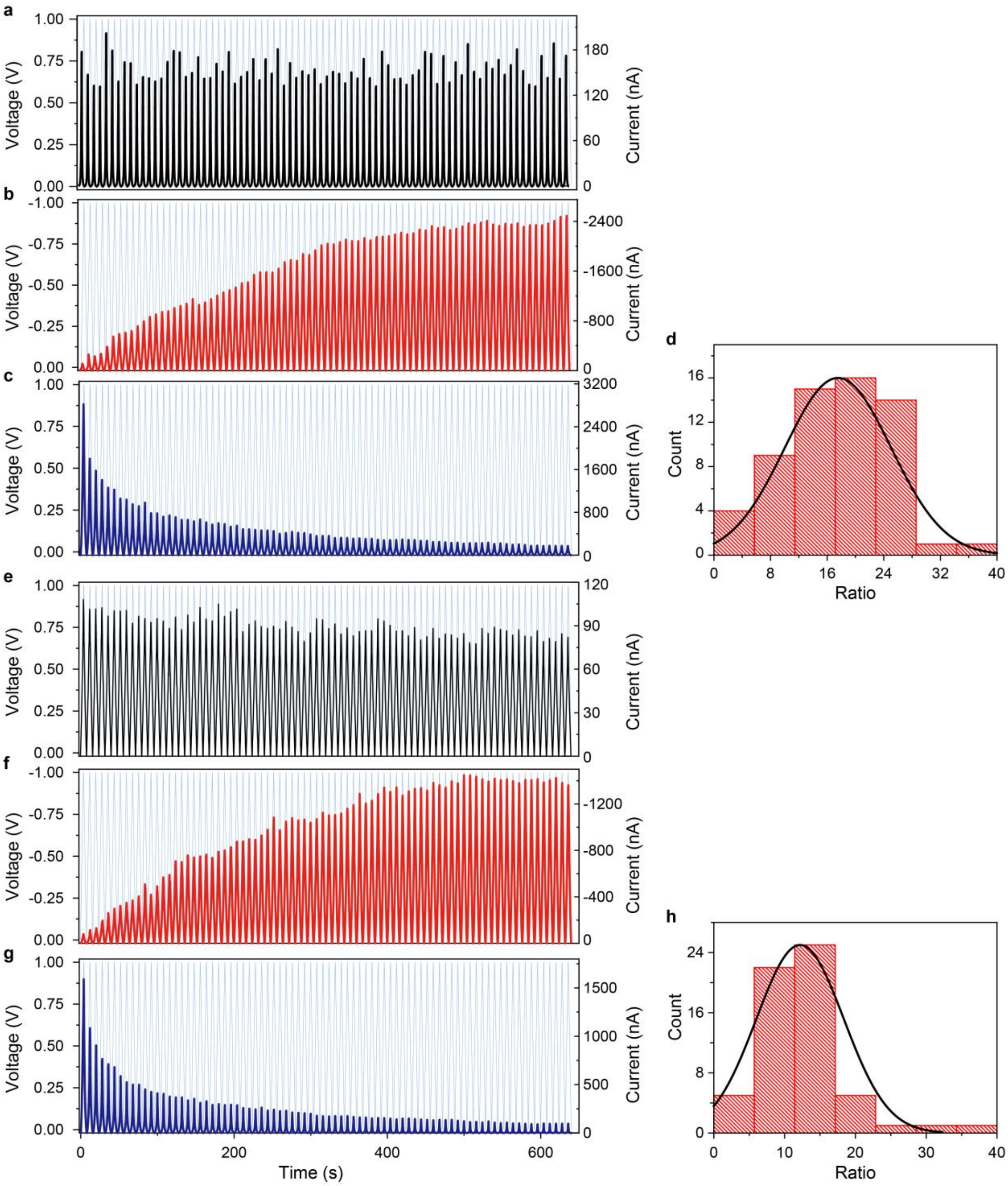

Fig. S12 | Electrical characteristics of the Ag/AgO$_x$//C(GABA)(GABA)D(GABA)(GABA)D//GaO$_x$/EGaIn and Ag/AgO$_x$//HS-PEG8-CH_2CH_2COOH//GaO$_x$/EGaIn devices. **a** to **c** (**e** to **g**), Current signals (*black*, *red*, and *blue* curves) recorded by consecutively placing 80 positive **a** (**e**), negative **b** (**f**), and positive **c** (**g**), triangle pulses (*light blue* curves, amplitude, 1 V, width, 8 s) on a typical

Ag/AgO$_x$//C(GABA)(GABA)D(GABA)(GABA)D//GaO$_x$/EGaIn (Ag/AgO$_x$//HS-PEG8-CH$_2$CH$_2$COOH//GaO$_x$/EGaIn) device. The bottom Ag/AgO$_x$ electrode is grounded. **d** and **h**, The histograms with a Gaussian fit of the current change ratio of 60 Ag/AgO$_x$//C(GABA)(GABA)D(GABA)(GABA)D//GaO$_x$/EGaIn and Ag/AgO$_x$//HS-PEG8-CH$_2$CH$_2$COOH//GaO$_x$/EGaIn devices under sequences of 80 triangle pulses. The current change ratio is calculated according to Ratio = I_{80}/I_1, where I_{80} (I_1) is the measured current of the 80th (1st) pulse.

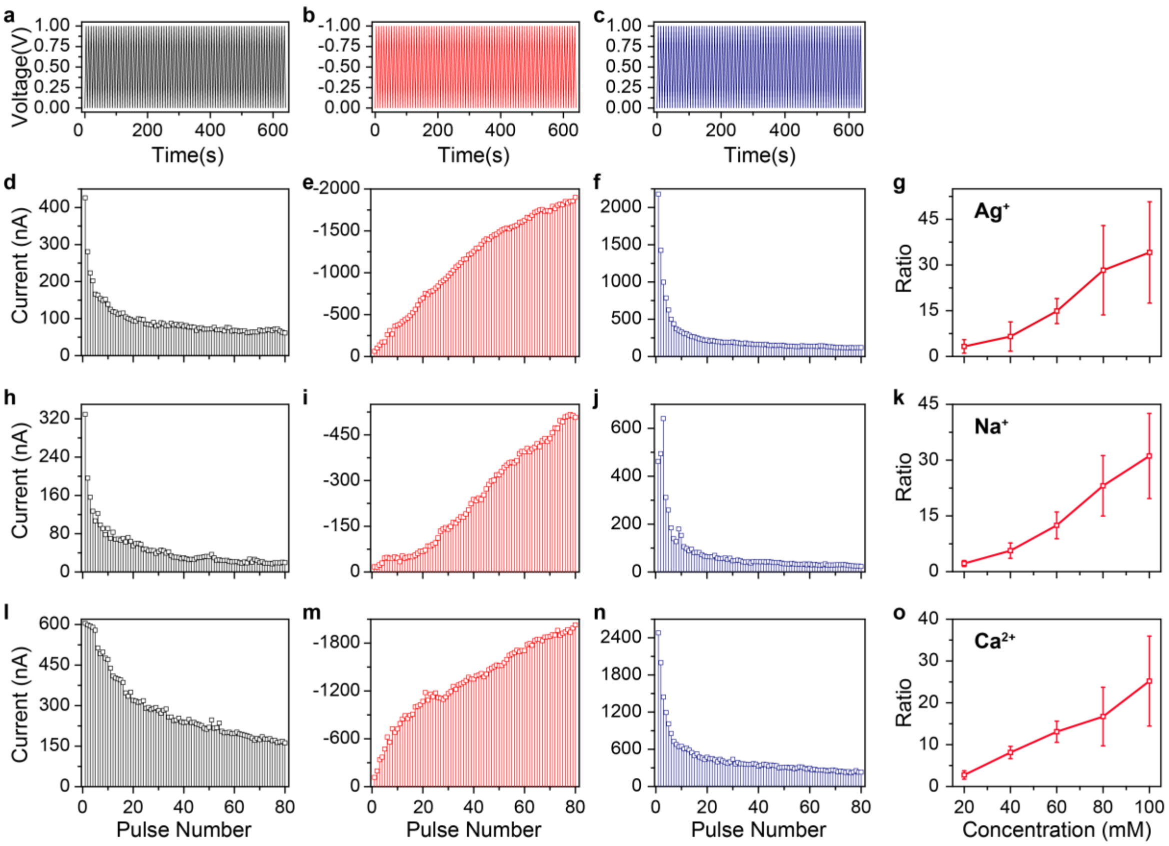

Fig. S13 | Electrical characteristics of the Ag//CAAAAKAAAAK//GaO$_x$/EGaIn devices doped with Ag^+, Na^+, and Ca^{2+} cations. Sequences of 80 positive **a**, negative **b**, and positive **c**, triangle pulses (amplitude, 1 V, width, 8 s) are consecutively placed on the junctions. The

bottom Ag electrode is grounded. **d** to **f** (**h** to **j** and **l** to **n**), The measured current peaks of a typical Ag//CAAAAKAAAAK//GaO_x/EGaIn device doped with Ag^+ (Na^+ and Ca^{2+}) under sequences of 80 triangle pulses shown in (a) to (c). **g** (**k** and **o**), The dependence of the current change ratio on the concentrations of Ag^+ (Na^+ and Ca^{2+}) cations. Each data point is acquired from ten devices. The current change ratio is calculated according to Ratio = I_{80}/I_1, where I_{80} (I_1) is the measured current of the 80th (1st) pulse. The solid lines are guides to the eye.

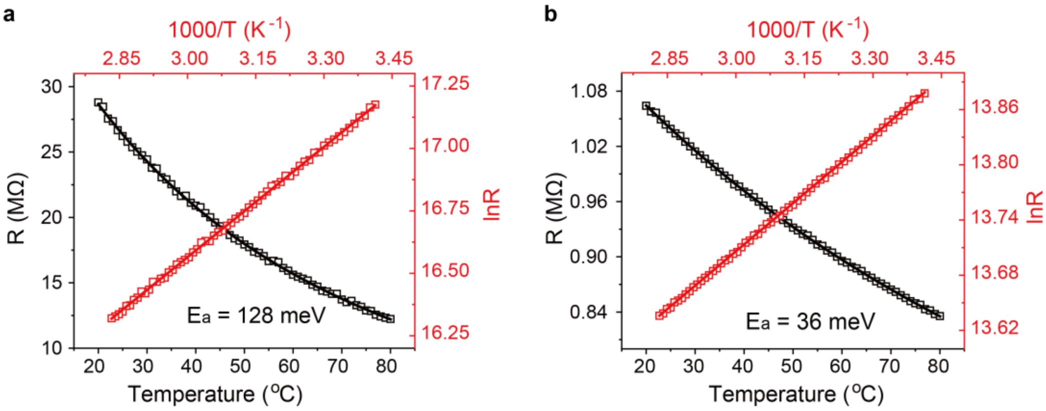

Fig. S14 | Temperature-dependent electrical characteristics. **a** and **b**, R *vs* temperature (*black* markers, *bottom* x-axis, and *left* y-axis) and Ln(R) *vs* 1000/T (*red* markers, *top* x-axis, and *right* y-axis) curves of the Ag//CAAAAKAAAAK//GaO_x/EGaIn device in the absence and presence of Ag^+ cations respectively. The curves are fitted by using the Arrhenius equation $R = R_\infty * \exp(E_a/k_B T)$, where R_∞ denotes resistance at $T = \infty\, K$, k_B is the Boltzmann constant, and E_a is the activation energy. In addition, the E_a can be evaluated by the relation $E_a = \frac{(\Delta G-\lambda)^2}{4\lambda}$ where the ΔG is the free energy difference between the two hopping states.

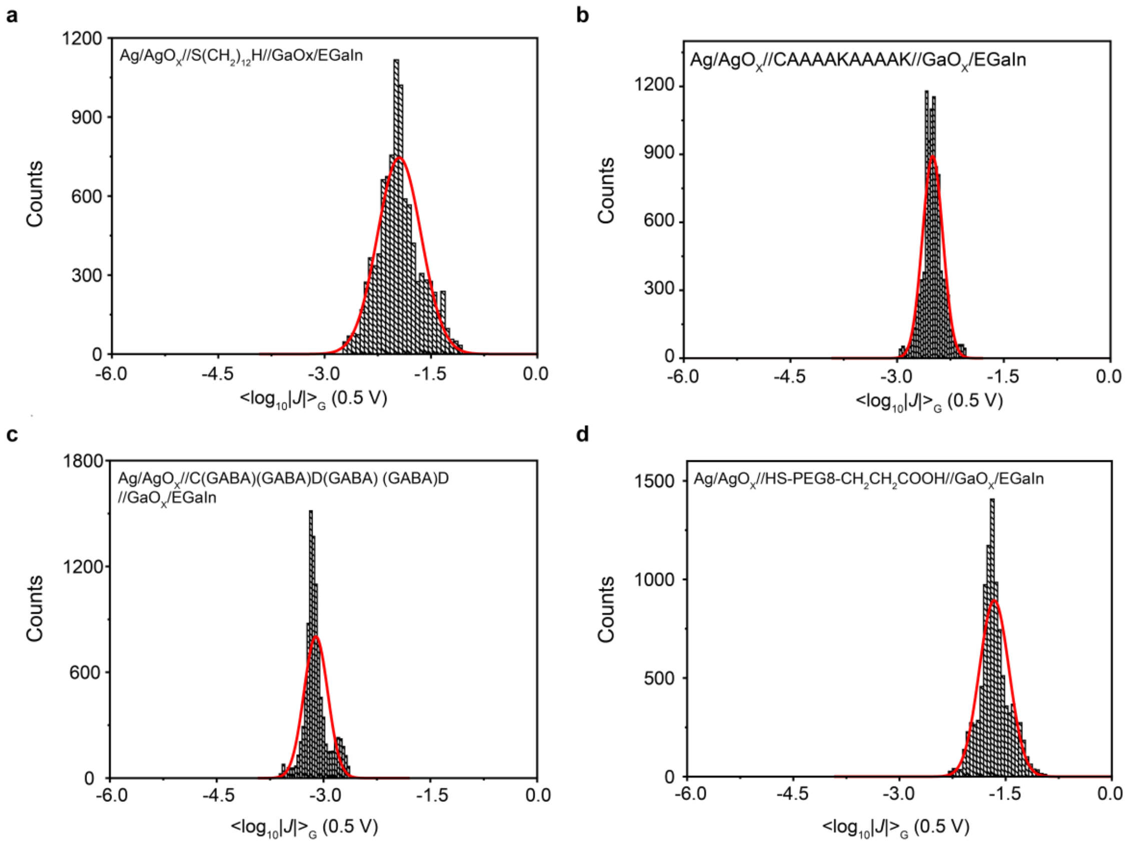

Fig. S15 | *J*(V) measurements with the voltages between 0 and 1 V of different molecular junctions. Histograms of $\log_{10}|J|$ at 0.5 V with the gaussian fits (*red* curves) of the **a**, Ag/AgO$_x$//HS-(CH$_2$)$_{12}$H//GaO$_x$/EGaIn, **b**, Ag/AgO$_x$//CAAAAKAAAAK//GaO$_x$/EGaIn, **c**, Ag/AgO$_x$//C(GABA)(GABA)D(GABA)(GABA)D//GaO$_x$/EGaIn, and **d**, Ag/AgO$_x$//HS-PEG8-CH$_2$CH$_2$COOH//GaO$_x$/EGaIn devices.

Table S1 | Statistical results of <$\log_{10}|J|_G$> at 0.5 V of the Ag/AgO_x//HS-$(CH_2)_{12}$H//GaO_x/EGaIn, Ag/AgO_x//CAAAAKAAAAK//GaO_x/EGaIn, Ag/AgO_x//C(GABA)(GABA)D(GABA)(GABA)D//GaO_x/EGaIn, and Ag/AgO_x//HS-PEG8-CH_2CH_2COOH//GaO_x/EGaIn devices[3-6].

Device	No. of junctions	No. of broken junctions	No. of traces	Yield (%)	<$\log_{10}J$>$_G$ (0.5 V) $\pm\sigma_{log}$
Ag/AgO_x//HS-$(CH_2)_{12}$H//GaO_x/EGaIn	74	60	9600	81	-1.95±0.31
Ag/AgO_x//CAAAAKAAAAK//GaO_x/EGaIn	81	60	9600	74	-2.50±0.14
Ag/AgO_x//C(GABA)(GABA)D(GABA)(GABA)D//GaO_x/EGaIn	77	60	9600	78	-3.11±0.17
Ag/AgO_x//HS-PEG8-CH_2CH_2COOH//GaO_x/EGaIn	82	60	9600	73	-1.66±0.21

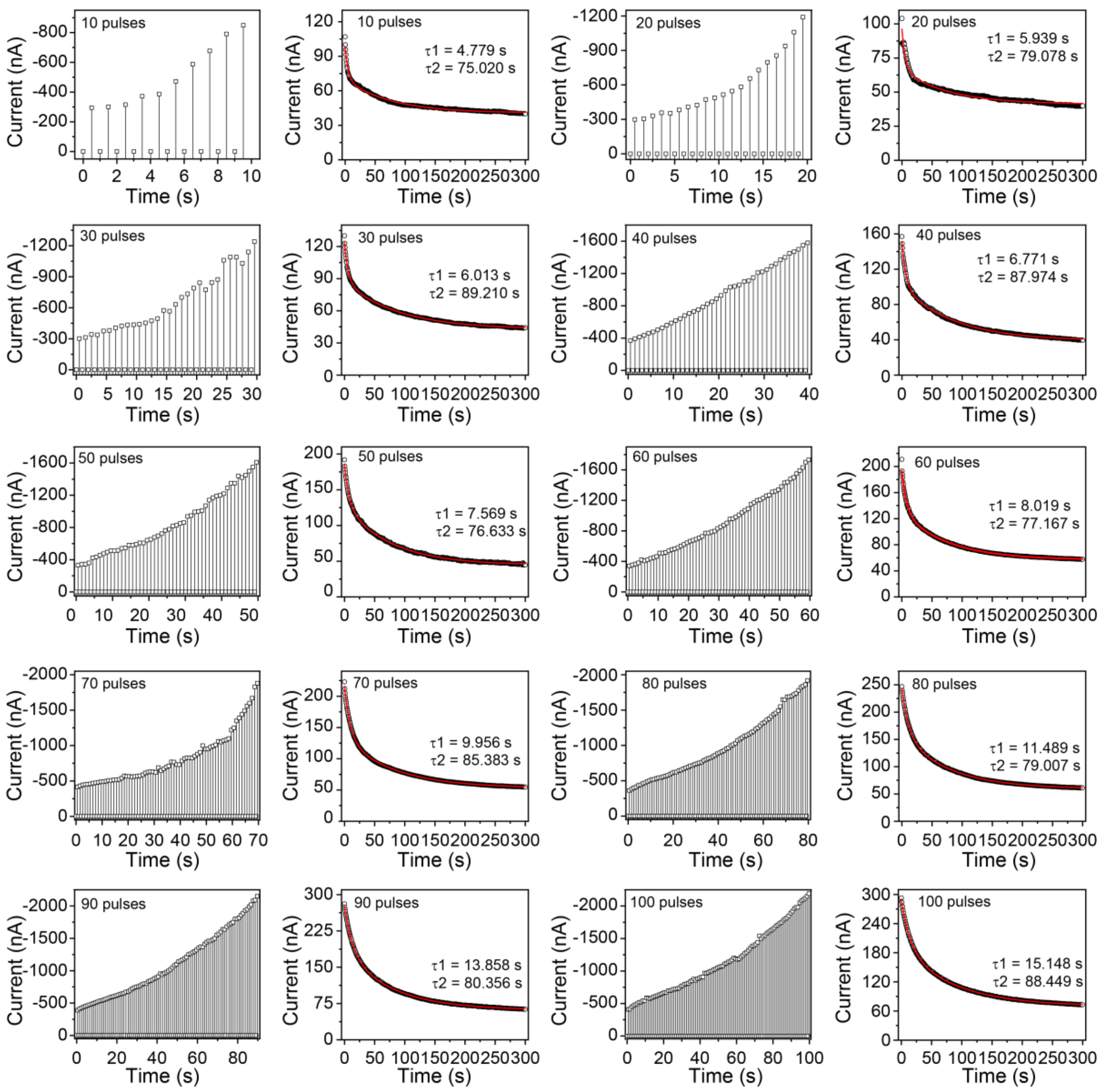

Fig. S16 | Potentiation current and current decay curves under pulses. The measured current of Ag/AgO_x//CAAAAKAAAAK//GaOx/EGaIn molecular synapse as potentiated by sequences of square pulses (amplitude, -1 V). The current decay curves are also recorded after potentiated (read bias, 0.15 V). In addition, the decay of the current (*red* solid lines) is fitted by using a double exponential function $I = a * \exp(-t/\tau_1) + b * \exp(-t/\tau_2) + c$ in which the time constant τ_1 and τ_2 correspond to the fast and slow decaying process.

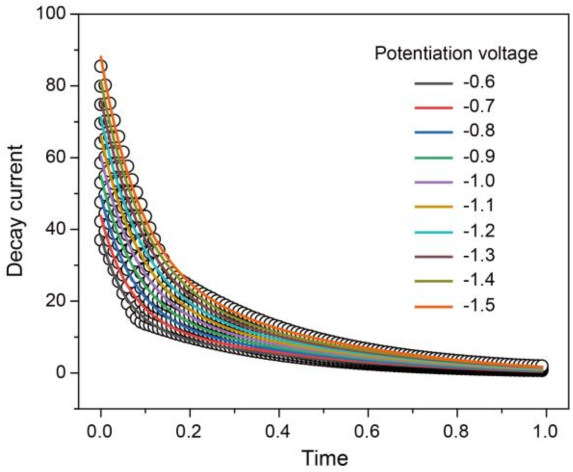

Fig. S17 | Simulated current decay. The simulated current decay curves after potentiated with the voltage from -0.6 to -1.5 V (*hollow* circle). The fitted solid lines are obtained by using a double exponential function $I = a * \exp(-t/\tau_1) + b * \exp(-t/\tau_2) + c$.

Table S2 | The hopping parameters for the two cases.

	λ (meV)	Δ (meV)	V (meV)	E_{a_Theory} (meV)	E_{a_Exp} (meV)
w/o Ag^+	267.9	471.8	235.9	250.3	128
w/ Ag^+	705.4	-45.0	243.0	154.5	36

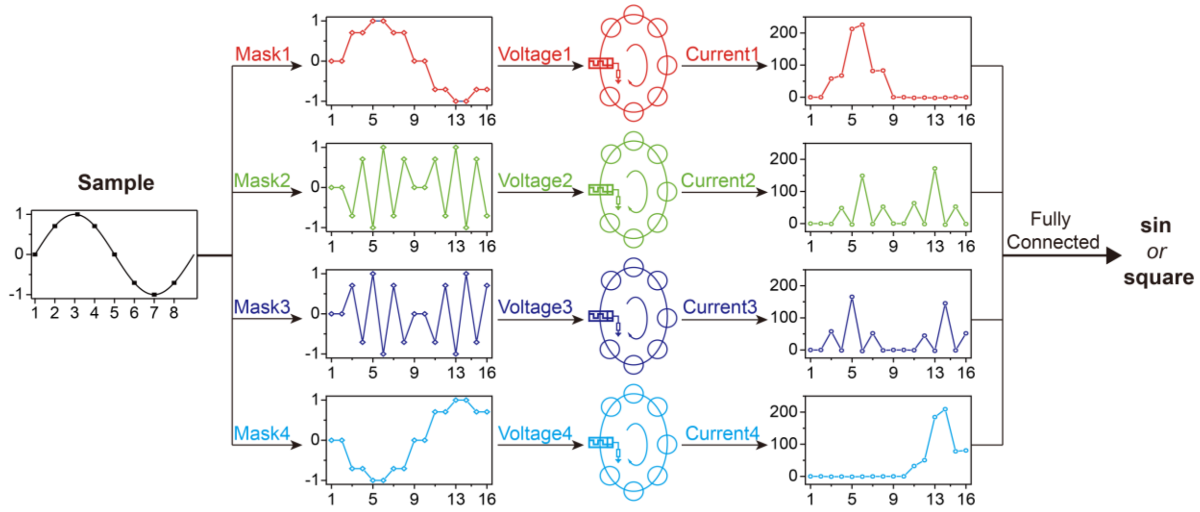

Fig. S18 | Parallel dynamic memristor-based reservoir computing system for the waveform recognition task. The system recognizes sine and square waveforms through 4 steps sampling input waveform, transforming sampled points, placing voltage pulses to a memristor, and calculating output categories by a linear combination of current vectors. The output weights are trained by linear regression.

SI references

1 Firet, N. J. *et al.* Operando EXAFS study reveals presence of oxygen in oxide-derived silver catalysts for electrochemical CO_2 reduction. *J. Mater. Chem. A* **7**, 2597-2607 (2019).

2 Rebelo, R., Calderon, S. V., Fangueiro, R., Henriques, M. & Carvalho, S. Influence of oxygen content on the antibacterial effect of Ag-O coatings deposited by magnetron sputtering. *Surf. Coat. Technol.* **305**, 1-10 (2016).

3 Baghbanzadeh, M. *et al.* Charge tunneling along short oligoglycine chains. *Angew. Chem. Int. Ed.* **54**, 14743-14747 (2015).

4 Chen, X. *et al.* Large increase in the dielectric constant and partial loss of coherence increases tunneling rates across molecular wires. *ACS Appl. Mater. Interfaces* **12**, 45111-45121 (2020).

5 Wan, A. *et al.* Arrays of high quality SAM-based junctions and their application in molecular diode based logic. *Nanoscale* **7**, 19547-19556 (2015).

6 Wan, A., Jiang, L., Sangeeth, C. S. S. & Nijhuis, C. A. Reversible soft top-contacts to yield molecular junctions with precise and reproducible electrical characteristics. *Adv. Funct. Mater.* **24**, 4442-4456 (2014).
